# Long Non-coding RNA HOTTIP Promotes CCL3 Expression and Induces Cartilage Degradation by Sponging miR-455-3p

**DOI:** 10.3389/fcell.2019.00161

**Published:** 2019-08-23

**Authors:** Guping Mao, Yan Kang, Ruifu Lin, Shu Hu, Ziji Zhang, Hongyi Li, Weiming Liao, Zhiqi Zhang

**Affiliations:** ^1^Department of Joint Surgery, First Affiliated Hospital of Sun Yat-sen University, Guangzhou, China; ^2^Department of Orthopaedic Surgery, First Affiliated Hospital of Sun Yat-sen University, Guangzhou, China

**Keywords:** HOTTIP, osteoarthritis, cartilage, miR-455-3p, CCL3 chemokine

## Abstract

Long non-coding RNAs (lncRNAs) play pivotal roles in diseases such as osteoarthritis (OA). However, knowledge of the biological roles of lncRNAs is limited in OA. We aimed to explore the biological function and molecular mechanism of HOTTIP in chondrogenesis and cartilage degradation. We used the human mesenchymal stem cell (hMSC) model of chondrogenesis, in parallel with, tissue biopsies from normal and OA cartilage to detect HOTTIP, CCL3, and miR-455-3p expression *in vitro*. Biological interactions between HOTTIP and miR-455-3p were determined by RNA silencing and overexpression *in vitro*. We evaluated the effect of HOTTIP on chondrogenesis and degeneration, and its regulation of miR-455-3p via competing endogenous RNA (ceRNA). Our *in vitro* ceRNA findings were further confirmed within animal models *in vivo*. Mechanisms of ceRNAs were determined by bioinformatic analysis, a luciferase reporter system, RNA pull-down, and RNA immunoprecipitation (RIP) assays. We found reduced miR-455-3p expression and significantly upregulated lncRNA HOTTIP and CCL3 expression in OA cartilage tissues and chondrocytes. The expression of HOTTIP and CCL3 was increased in chondrocytes treated with interleukin-1β (IL-1β) *in vitro*. Knockdown of HOTTIP promoted cartilage-specific gene expression and suppressed CCL3. Conversely, HOTTIP overexpression reduced cartilage-specific genes and increased CCL3. Notably, HOTTIP negatively regulated miR-455-3p and increased CCL3 levels in human primary chondrocytes. Mechanistic investigations indicated that HOTTIP functioned as ceRNA for miR-455-3p enhanced CCL3 expression. Taken together, the ceRNA regulatory network of HOTTIP/miR-455-3p/CCL3 plays a critical role in OA pathogenesis and suggests HOTTIP is a potential target in OA therapy.

## Introduction

Osteoarthritis (OA) is the most widespread chronic joint disease, characterized by progressive destruction of cartilage integrity, thickening of subchondral bone, osteophyte formation, and joint-space narrowing, rendering it a leading cause of disability (Goldring and Goldring, 2007). Unfortunately, no effective disease-modifying OA drugs (DMOADs) exist, resulting in serious socioeconomic burdens (Tonge et al., 2014). Chemokines are tiny chemotactic cytokines that play vital roles in inflammatory diseases such as rheumatoid arthritis (RA) and OA (Gerard and Rollins, 2001; Haringman et al., 2004). Proinflammatory factors derived from the synovium and chondrocytes, such as TNF-α and IL-1β, stimulate chemokine expression in the initial and developmental progression of OA (Gerard and Rollins, 2001). Chemokines maintain similar polypeptide sequences, weighing 8–12 kD, with two major subclasses characterized by the presence of either adjacently conserved cysteine residues (CC) or residues separated by a non-specific, intervening amino-acid (CXC) (Gerard and Rollins, 2001; Haringman et al., 2004). Numerous studies report chemokines that play important roles in activating catabolic pathways and chondrocyte hypertrophy (Mazzetti et al., 2004; Chauffier et al., 2012; Scanzello, 2017). For example, CCL2 increased MMP-3 expression and promoted collagen and aggrecan proteolysis in OA cartilage chondrocytes, confirmed in an animal model (Borzi et al., 2000). Previous studies revealed that IL-1β-dependent induction of CCL3 expression in primary human chondrocytes (PHCs) promotes cartilage degradation (Sandell et al., 2008; Zhang et al., 2010) and that plasma CCL3 could be a potential serum biomarker in knee degeneration (Zhao et al., 2015). Further research has demonstrated the essential role of CCL3 in the development of joint arthritis in CCL3-null mice (Chintalacharuvu et al., 2005).

It is widely accepted that non-coding RNA (ncRNA) plays a pivotal role in chondrogenesis and OA progression (Barter and Young, 2013; Peffers et al., 2018). Non-coding RNAs include microRNAs (miRNAs) and long non-coding RNAs (lncRNA). MiRNAs (∼22-nt) are robust regulators of post-transcriptional gene expression with a high affinity for the 3′ untranslated region, ultimately carrying out their function through recruitment of protein complexes that block ribosomal translation and contribute to degradation of the poly-adenylated tail (Bartel, 2004). Previously, we reported a 2.973-fold upregulation of miR-455-3p upon differentiation of human adipose-derived stem cells into chondrocytes (Zhang et al., 2012) and provided evidence that miR-455-3p is an important regulator of chondrogenesis and cartilage degeneration via the inhibition of RUNX2/HDAC2/HDAC8/DNMT3A (Zhang et al., 2015; Chen et al., 2016; Sun H. et al., 2018). Although studies of miRNAs have dominated the field of RNA biology in recent years, accumulating evidence shows that lncRNAs (>200-nt) are central regulators of biological processes, including those involved in the pathology of OA (Liu et al., 2014; Xing et al., 2014; Pearson et al., 2016); they regulate gene expression at both the transcriptional and post-transcriptional levels (Yang et al., 2014). One study revealed a greater than 10-fold upregulation in expression of HOTTIP in OA compared to that in normal chondrocytes (Kim et al., 2013). Moreover, HOTTIP has been identified as a ceRNA (competing endogenous RNA) that protects integrin-α1 messenger RNA (mRNA) from miR-101-mediated degradation (Kim et al., 2013). Long non-coding RNA Growth Arrest-Specific 5 (GAS5) has also been identified as a ceRNA that promotes MMPs by sponging miR-21 during OA (Song et al., 2014). Previous studies identified HOTTIP as an important regulator in OA and small cell lung cancer chemoresistance and osteoblast differentiation (Kim et al., 2013; Sun Y. et al., 2018; Da et al., 2019).

However, the molecular functions and mechanisms of HOTTIP in chondrogenesis and cartilage degradation have not been fully elucidated. In our current study, based on bioinformatics software (TargetScan and RNA22^1^) and various experimental approaches, we suggest that HOTTIP functions as a ceRNA to regulate the expression of CCL3 and the cartilage-specific genes via sponging miR-455-3p in cartilage development and degradation.

## Materials and Methods

All experimental protocols were approved by the Ethics Committee of the First Affiliated Hospital of Sun Yat-sen University and the Declaration of Helsinki (2000). All participants signed off on informed consent.

### Human Mesenchymal Stem Cell Culture and Induction of Chondrogenesis

Bone marrow samples were derived from the iliac crest aspiration of six normal donors (three males and three females; age range: 31–39 years). Human mesenchymal stem cells (hMSCs) were isolated with density-gradient centrifugation and cultured as described previously (Meng et al., 2014). All hMSCs were used at passage three to induce hMSC chondrogenesis by micromass culture, as previously described (Zhang et al., 2012). Samples were collected for experiments at selected time points.

### Cartilage Cell Isolation and Culture

Cartilage samples were gathered from eight donors (five males and three females; age range: 55–65 years) who underwent total hip replacement surgery due to fractures of the femoral neck without previous history of OA or RA, For OA chrondrocytes, biopsies were taken from eight participants (four males and four females; age range: 56–64 years) undergoing total knee arthroplasty (TKA) because of knee joint OA. According to the Outerbridge classification scale (Outerbridge, 1961), the grade II and III cartilages were collected and pooled as degraded cartilage. PHCs were isolated and cultured from cartilage as described previously (Sun H. et al., 2018). All PHCs were used at passage one.

### RNA Extraction, Reverse Transcription, and RT-qPCR

With miRNA Mini Kit (Qiagen, Hilden, Germany), total RNA from cells and cartilage samples were extracted following manufacturer’s instructions. Next, cDNA was synthesized from miRNA and mRNA using a Mir-X^TM^ miRNA First-Strand Synthesis Kit (Takara Bio Inc., Shiga, Japan) and a PrimeScript^TM^ RT Master Mix (Takara), respectively. RT-qPCR of target genes was performed with SYBR^®^ Premix Ex Taq^TM^ II (Takara) and a CFX96 real-time qPCR instrument (Bio-Rad, Hercules, CA, United States). Transcript levels were normalized to glyceraldehyde 3-phosphate dehydrogenase (GAPDH) for mRNA and U6 RNA for miRNA, qPCR primer sequences can be found in Supplementary Table 1. The mRQ 3′ Primer (Clontech) was used as the reverse primer for miRNA-455-3p. Relative gene expression was calculated using the 2^–Δ^ ^Δ^ ^*Ct*^ method, and each experiment was performed in triplicate.

### Western Blot Analysis

Western blotting protocols were followed as previously described (Mao et al., 2017). Total protein of hMSCs and PHCs was isolated. 25 μg of each protein sample was separated by sodium dodecyl sulfate-polyacrylamide gel electrophoresis (SDS-PAGE) and transferred to polyvinylidene difluoride membranes (Millipore, Bedford, MA, United States). Membranes were incubated with primary antibodies specific for RUNX2, MMP3 [1:1,000 dilution, cell signaling technology (CST), Boston, MA, United States]; COL2A1, aggrecan, MMP13 (1:2,000 dilution, Abcam, Cambridge, MA, United States); SOX9 (1:2000 dilution, Millipore, MA, United States); and β-actin (1:3,000 dilution, CST). Appropriate secondary antibodies (1:3,000 dilution, CST) were used to incubate the blots at room temperature for 1 h, after which they were developed with an ECL chemiluminescence Kit (Millipore).

### Animal and Histology Staining

All procedures were approved by the ethical committee of the First Affiliated Hospital of Sun Yat-sen University (IRB: 2014C-028). The mmu-miR-455-3p global knockout mice were generated using a transcription activator-like effector nuclease (TALEN) system – refer to previous study for detailed protocol (Sun H. et al., 2018). DNA sequencing analysis was performed by PCR. The wild-type (WT) (miR-455-3p +/+), heterozygous (miR-455-3p ±), and homozygous (miR-455-3p−/−) mice were bred through the mating of miR-455-3p ± male and female mice. In this study, pregnant miR-455-3p-deficient and WT C57B/L6 mice were obtained from the Animal Center of Sun Yat-sen University. Forelimbs were harvested from embryonic mice at 16.5 days post-coitum (E16.5). Additionally, we harvested the knees of 10-month-old miR-455-3p-deficient and C57B/L6-WT mice. Samples were fixed in 4% paraformaldehyde (Sigma–Aldrich, St. Louis, MO, United States) for 24hrs, decalcified in ethylenediaminetetraacetic acid (EDTA) for 2 weeks, embedded in paraffin, and cut into 5-μm sections that were deparaffinized, rehydrated, and stained with Safranin O/Fast Green. COL2A1, aggrecan, MMP3, MMP13 and CCL3 expression was analyzed by immunohistochemistry, as described previously (Chen et al., 2016). For miR-455-3p and HOTTIP *in situ* hybridization, tissue samples were dehydrated with a graded series of ethanol, embedded in paraffin, and cut into 5-μm sections. Sections were incubated in 10 μg/mL Proteinase K (Promega, WI, United States) at 37°C for 20 min. The sections were incubated with miR-455-3p (mmu-miR-455-3p probe: 5′-GTGTATATGCCCATGGACTGC-3′)- or HOTTIP (mmu-HOTTIP probe: 5′-TGGCATAATGACTGTATTTCACTACGCT TG-3′)-specific probes (Exiqon, Denmark) at 56 °C for 1 h after dehydration. Endogenous alkaline phosphatase was blocked at room temperature for 15 min, and anti-Digoxigenin-AP (1: 500, Roche, United States) was applied at room temperature for 1h and the following step as described in our previous study (Zhang et al., 2015).

### Transfection of Small-Interfering RNA (siRNA) Molecules, miR-455-3p Mimics and Inhibitors, and Plasmids

HMSCs and PHCs were transfected with an agomir (50 nM) or antagomir (100 nM) (RiboBio, Guangzhou, China) of miR-455-3p, pcDNA3.1-HOTTIP (500 ng) plasmids, or siHOTTIP (100 nM) (ObiO Technology, Shanghai, China). PHCs were also transfected with siCCL3 (100 nM) or siNC (RiboBio). Transfections were performed using Lipofectamine 2000 Reagent (Invitrogen) following the manufacturer’s protocol. For hMSC chondrogenic differentiation by micromass culture, hMSCs monolayers were transfected twice, the day after plating and three days after plating, respectively.

### Luciferase Reporter Assay

The psiCHECK2 luciferase vector (Promega, WI, United States) was used for the dual luciferase assays. HOTTIP and CCL3 were cloning on the Renilla luciferase downstream. Recombinant plasmids of psiCHECK2.0-H-HOTTIP-WT (NR_037843.3), psiCHECK2.0-H-CCL3-WT 3′-UTR(NM_002983), or corresponding mutant type (Mut) were constructed by ObiO Technology (Shanghai, China). HEK293 cells (1.2 × 10^4^ cells per well) were plated in a 96-well plate and co-transfected by Lipofectamine 2000 with 100 nM of either miR-455-3p or nonsense miRNA of either recombinant plasmids(0.2 ug/well) or corresponding mutants(0.2 ug/well). Luciferase activity was measured 48 h post-transfection with the Dual-Luciferase^®^ Reporter Assay System (Promega, Madison, WI, United States), according to the manufacturer’s instructions.

### RNA Pull-Down Assay and Mass Spectrometry

The HOTTIP plasmid were transcribed and biotin-labeled *in vitro* with Bio-16-UTP (Life Technologies) using a TranscriptAid T7 High Yield Transcription Kit (Life Technologies). Protein–RNA interactions were carried out using a Pierce Magnetic RNA-Protein Pull-Down Kit (Life Technologies) with the lysates of chondrocytes. Then, the retrieved proteins were detected by western blot analysis or resolved by in-gradient gel electrophoresis followed by mass spectrometry (MS) identification (Xu et al., 2017).

### RNA Immunoprecipitation (RIP) Based on AGO2

PHCs were transfected with siHOTTIP or siNC. After 48 h, RNA Immunoprecipitation (RIP) experiments were performed using transfected PHCs with an anti-AGO2 antibody (Millipore, MA, United States) and the Magna RIP^TM^ RNA-Binding Protein Immunoprecipitation Kit (Millipore). Briefly, chondrocytes were lysed by RIP lysis buffer. Then, 100-μL cell extract was incubated with RIP buffer containing magnetic beads conjugated with human anti-Ago2 antibody (Millipore) or negative control (normal mouse IgG, Millipore). After the antibody was recovered by protein A/G beads, qRT-PCR was performed to detect HOTTIP and CCL3 mRNA level in the precipitates.

### Collagenase VII-Induced OA Mouse Model

All procedures were approved by the Animal Research Committee of the First Affiliated Hospital of Sun Yat-sen University. Twelve-week-old male C57B/L6 mice were randomly divided into five groups: normal group (*n* = 5); agomir-455-3p group (*n* = 5); pcDNA3.1-HOTTIP group (*n* = 5); pcDNA3.1-HOTTIP + agomir-455-3p group (*n* = 5); and OA group (*n* = 5). The following experiments were conducted in a sterile environment in order to avoid joint infection. On day 0, mice in the agomir-455-3p, pcDNA3.1-HOTTIP, pcDNA3.1-HOTTIP + agomir-455-3p, and OA groups were OA-induced with collagenase VII (12 U of collagenase VII in 8 μL saline, produced by *Clostridium histolyticum*; Sigma–Aldrich) as described previously (van Osch et al., 1993; Johnson et al., 2012). On days 7, 14, and 21, the agomir-455-3p, pcDNA3.1-HOTTIP, and pcDNA3.1-HOTTIP + agomiR-455-3p groups were injected with 15 μL agomir-455-3p (100 nmol), 15 μL pcDNA3.1-HOTTIP (1.5 μg/μL), or 15 μL pcDNA3.1-HOTTIP + agomir-455-3p, respectively. Mice in the normal groups were injected with 15 μL saline. On day 56, mice were euthanized and the joints were stained for further analysis.

### Statistical Analysis

Each experiment was performed at least three times. Experiments data are showed as means ± standard deviations (SD) Student’s *t*-tests and Mann–Whitney U-tests were used to determine differences between groups. One-way analysis of variance (ANOVA) and Kruskal–Wallis tests were performed for multiple group comparisons. *P* values less than 0.05 were considered statistically significance. Data analysis was performed with SPSS software.

## Results

### Expression Patterns of miR-455-3p, HOTTIP and CCL3 During hMSC Chondrogenesis

**FIGURE 1:**
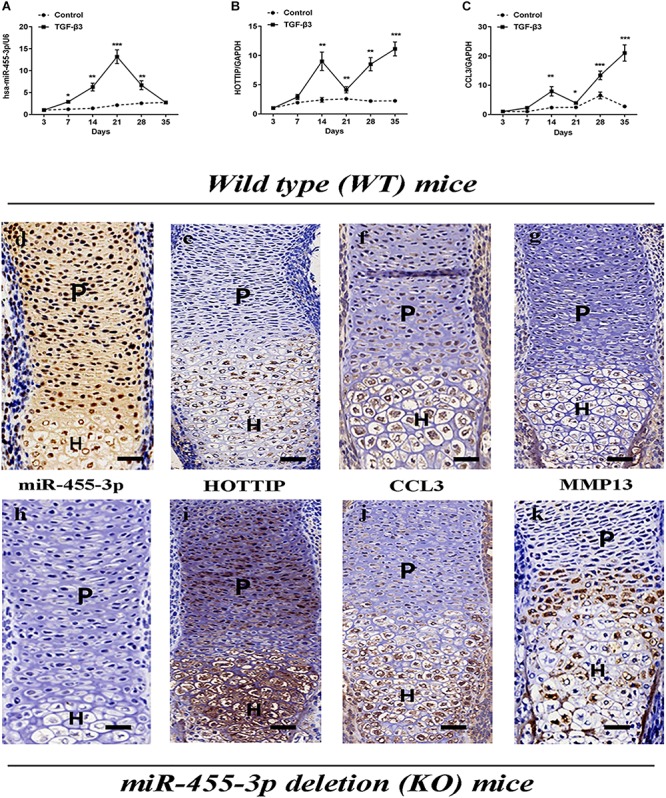
Analysis of the relative expression levels of miR-455-3p, HOTTIP and CCL3 during the chondrogenesis of hMSCs and in forelimbs harvested from WT mice embryos or miR-455-3p-deficient (KO) mice embryos. MSCs were induced to chondrogenesis with TGF-β3 for 3, 7, 14, 21, 28, and 35 days as indicated (solid lines). Gene expression of miR-455-3p **(A)**, HOTTIP **(B)** and CCL3 **(C)** were determined by RT-qPCR. MSCs cultured without TGF-β3 at corresponding time points served as negative controls (dashed lines). U6 and GAPDH expression levels were measured and used as internal controls for microRNA and mRNA expression, respectively. The data shown represent the mean ± SD of three independent experiments in samples from three different donors.^*^P < 0.05. *In situ* hybridization analysis of miR-455-3p **(D,H)** and HOTTIP **(E,I)** expression in tissue sections from the radius of mouse embryos were harvested at 16.5 days post-coitum. Sections were immunostained (brown) with antibodies specific to CCL3 **(F,J)** and MMP13 **(G,K)**, respectively. P, proliferating chondrocytes; H, hypertrophic chondrocytes; scale bar: 50 μm.

HMSCs were induced into chondrogenic lineage via micromass. We observed an upregulation of miR-455-3p at 7 days that peaked at 21 days, followed by a reduction at days 28 and 35 (Figure 1A). Inverse expression patterns in HOTTIP, and CCL3 were found during the chondrogenic differentiation of hMSCs at 14–35 days (Figures 1B,C), suggesting that miR-455-3p may have an effect on HOTTIP and CCL3 expression.

### Expression of miR-455-3p, HOTTIP and CCL3 During Cartilage Development

It has been previously reported that proliferating and hypertrophic chondrocytes could be detected in E16.5 mouse limbs (Kim et al., 2010). In order to characterize our observed expression patterns of miR-455-3p, HOTTIP, and CCL3 during different stages of chondrocyte development *in vivo*, the forelimbs of miR-455-3p-deficient and C57B/L6-WT mice were isolated at E16.5 and subjected to *in situ* hybridization and immunohistochemistry analyses. It is important to note that while moderate to high levels of miR-455-3p expression were detected in proliferating chondrocytes, little to no miR-455-3p expression was observed in hypertrophic chondrocytes (Figures 1D,H). Conversely, HOTTIP, expression was detected in hypertrophic chondrocytes, with little to no expression observed in proliferating chondrocytes (Figures 1E,I). Compared to WT mice, the forelimbs of miR-455-3p-deficient mice showed higher MMP13 expression in hypertrophic zones (Figures 1J,K).

### HOTTIP Enhances the Expression of CCL3 by Sponging miR-455-3p in hMSCs During Chondrogenesis

**FIGURE 2:**
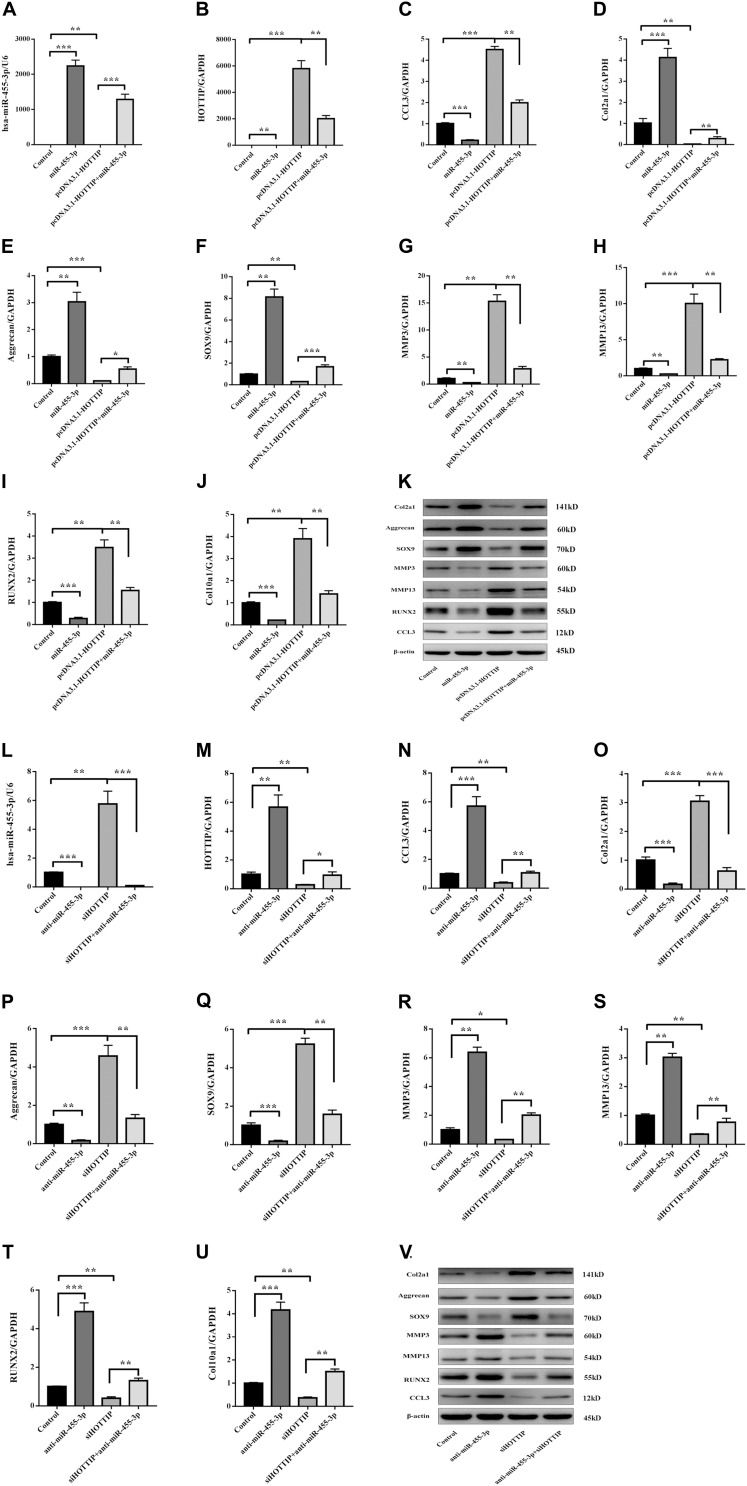
miR-455-3p and HOTTIP regulate the expression of CCL3 and cartilage-specific gene during hMSCs chondrogenesis. hMSCs were transfected with miR-control, miR-455-3p, pcDNA3.1-HOTTIP, pcDNA3.1-HOTTIP + miR-455-3p, anti-miR-control, anti-miR-455-3p, siHOTTIP, siHOTTIP + anti-miR-455-3p, respectively, and then induced to differentiate into chondrocytes for 14 days. The expression level of miR-455-3p **(A,L)** and HOTTIP **(B,M)** were estimated by RT-qPCR, while the expression levels of CCL3 **(C,K,N,V)** and Col2a1 **(D,K,O,V)**, Aggrecan **(E,K,P,V)**, SOX9 **(F,K,Q,V)**, MMP3 **(G,K,R,V)**, MMP13 **(H,K,S,V)**, RUNX2 **(I,K,T,V),** and COL10A1 **(J,K,U,V)** were estimated by RT-qPCR and western blotting. GAPDH and β-actin were used as endogenous controls. The data shown represent the mean ± SD of three independent experiments performed with samples from three different donors. ^*^p < 0.05, ^∗∗^*p* < 0.01, ^∗∗∗^*p* < 0.001.

To investigate whether HOTTIP enhances CCL3 expression by sponging miR-455-3p during chondrogenic differentiation, HOTTIP was either inhibited or overexpressed in hMSCs. These hMSCs were transfected with either miR-455-3p or anti-miR-455-3p, or pcDNA3.1 HOTTIP or siHOTTIP, and then differentiated into chondrogenic lineage for 14 days (Figure 2). Overexpression HOTTIP induced a significant increase CCL3, RUNX2, MMP3, and MMP13 and an evident reduction SOX9, COL2A1, and aggrecan expression. However, these changes in expression were partially abolished when miR-455-3p was co-transfected (Figures 2A–K). In contrast, inhibition HOTTIP induced a significant decrease CCL3, RUNX2, MMP3, and MMP13 with a significant increase in SOX9, COL2A1, and aggrecan expression. These effects were partially abolished when anti-miR-455-3p was co-transfected (Figures 2L–V). We found that HOTTIP enhanced the CCL3 expression in hMSCs chondrogenic differentiation and accelerated degeneration, while the effect can be partially prevented by miR-455-3p.

### Expression of miR-455-3p, HOTTIP and CCL3 in Normal and OA Cartilage

**FIGURE 3:**
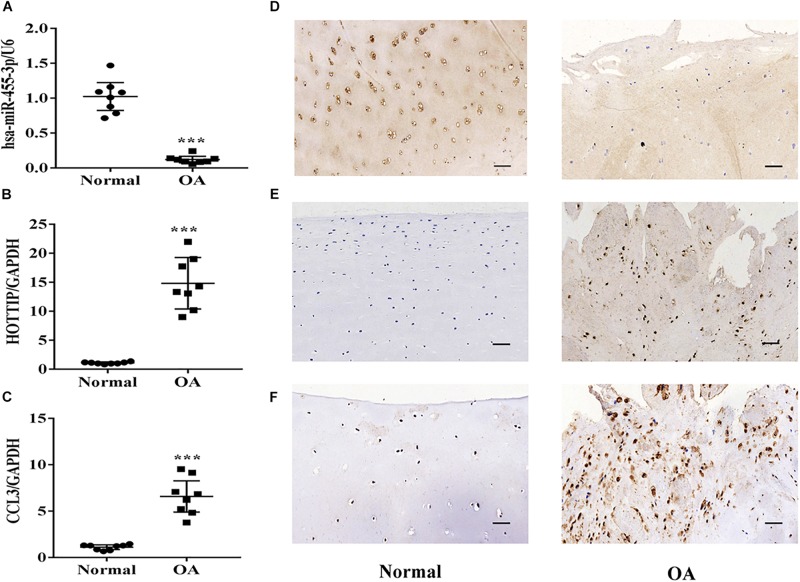
Expression of miR-455-3p, HOTTIP and CCL3 in normal and OA cartilage. Relative miR-455-3p, HOTTIP and CCL3 mRNA levels in normal and OA cartilages were determined by RT-qPCR **(A,B,C)**. U6 and GAPDH were used as endogenous controls. Each dot represents a value from a single experiment of one donor. The bar shows the mean and 95% confidence intervals of the values from six different donors per group. The miR-455-3p and HOTTIP expression levels were determined in normal cartilage and OA cartilage by *in situ* hybridization **(D,E)**. CCL3 protein levels in normal cartilage and OA cartilage were determined by immunohistochemistry **(F)**. Data shown are representative of results from six normal and OA cartilages. Scale bar: 50 μm. ^∗∗∗^*p* < 0.001.

To determine whether miR-455-3p or HOTTIP expression is altered during the progression of OA, we detected their expression levels in normal and OA cartilage by RT-qPCR, *in situ* hybridization, and immunohistochemistry. MiR-455-3p (Figures 3A,D) expression level was significantly downregulated in OA cartilage compared with normal cartilage, while the expression of HOTTIP and CCL3 (Figures 3B,C,E,F) was upregulated.

### miR-455-3p Regulates HOTTIP, CCL3, and Cartilage-Specific Gene Expression in Human Primary Chondrocytes

**FIGURE 4:**
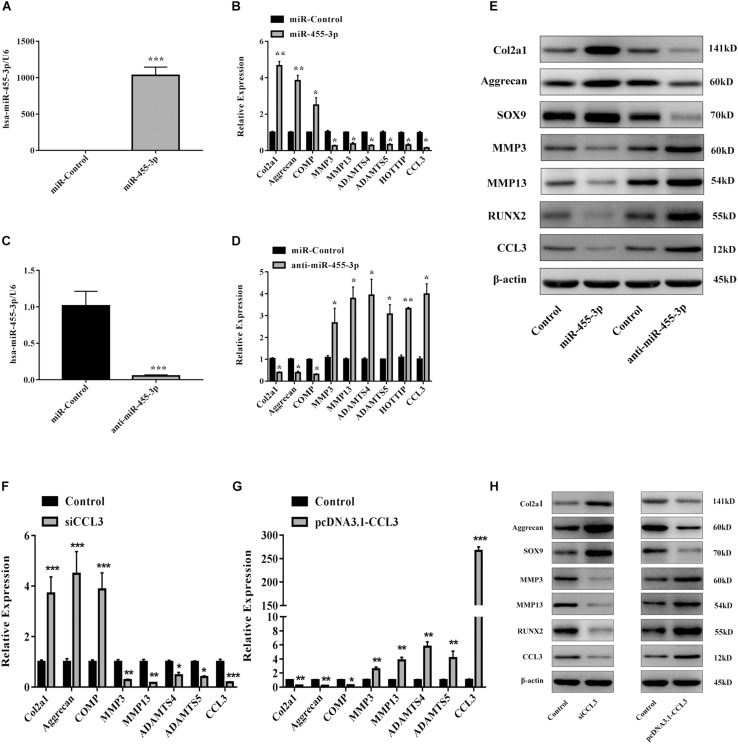
miR-455-3p regulates HOTTIP and CCL3 expression in PHCs and PHCs were transfected with siCCL3 or pcDNA3.1-CCL3. PHCs were transfected with 50 nM miR-control or miR-455-3p and 100 nM anti-miR-control or anti-miR-455-3p. After 48 h, **(A–D)** the relative expression levels of miR-455-3p, CCL3, HOTTIP, Col2a1, Aggrecan, COMP, ADAMTS-4, ADAMTS-5, MMP3 and MMP13 were estimated by RT-qPCR. **(E)** The protein levels of COL2A1, aggrecan, SOX9, MMP3, MMP13, and CCL3 were visualized by western blotting. And the gene and protein expression levels of COL2A1, aggrecan, COMP, MMP3, MMP13, ADAMTS-4, ADAMTS-5, and CCL3 were estimated by RT-qPCR **(F,G)** and western blotting **(H)**. U6, GAPDH, and β-actin were used as endogenous controls. The data shown represent the mean ± SD of at least three independent experiments. ^*^p < 0.05, ^∗∗^*p* < 0.01, ^∗∗∗^*p* < 0.001.

To assess whether miR-455-3p regulates HOTTIP and CCL3 expression in chondrocytes, we either inhibited or overexpressed miR-455-3p in PHCs. MiR-455-3p, miR-Control, anti-miR-455-3p, or anti-miR-Control were transfected into PHCs for 48 h. Overexpression of miR-455-3p decreased the expression of HOTTIP, CCL3, ADAMTS-4, ADAMTS-5, MMP3, and MMP13 and increased the levels of COMP, COL2A1, and aggrecan (Figures 4A,B). On the contrary, suppression of miR-455-3p with anti-miR-455-3p resulted in a significant increase in HOTTIP, CCL3, ADAMTS-4, ADAMTS-5, MMP3, and MMP13 and a corresponding decrease in COL2A1, COMP, and aggrecan (Figures 4C,D). Quantification of COL2A1, aggrecan, SOX9, RUNX2, CCL3, MMP3, and MMP13 proteins was determined by western blotting (Figure 4E). We found that miR-455-3p overexpression suppressed HOTTIP and CCL3 levels and enhanced cartilage-specific gene expression.

### CCL3 Suppresses Cartilage-Specific Gene Expression

Using a similar methodology geared at investigating the functionality of CCL3 in primary chondrocytes, siRNA was used to silence the levels of CCL3. PHCs were transfected with Control, siCCL3, and pcDNA3.1-CCL3. SiCCL3 increased cartilage-specific gene and protein expression and downregulated the levels of ADAMTS-4, ADAMTS-5, MMP3, and MMP13 (Figures 4F,H). Inversely, overexpression of CCL3 lead to downregulation of cartilage-specific gene expression and upregulated ADAMTS-4, ADAMTS-5, MMP3, and MMP13 (Figures 4G,H). This data indicates that CCL3 may play a key role in regulating cartilage degradation in PHCs.

### Inverse Expression Correlation Between HOTTIP and miR-455-3p in Response to IL-1β-Induced Human Chondrocytes

**FIGURE 5:**
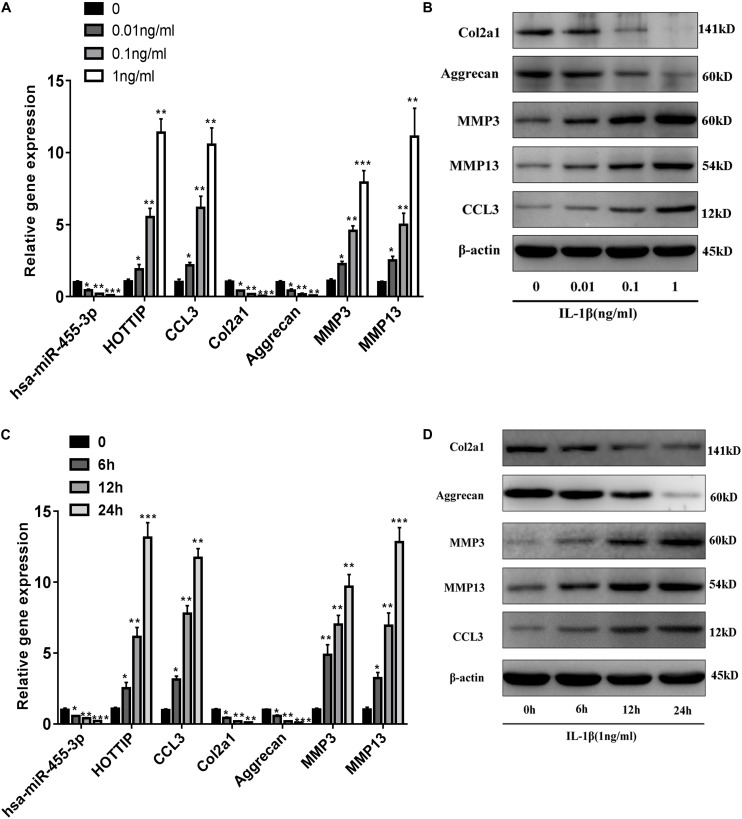
Expression of miR-455-3p, HOTTIP and CCL3 in IL-1β-stimulated human primary chondrocytes and an inverse correlation between IL-1 β-regulated HOTTIP, CCL3 and miR-455-3p expression in PHCs. PHCs were deprived of serum for 6 h, and then left untreated or treated with various concentrations (0, 0.01, 0.1 and 1 ng/ml) of IL-1β after 24 h **(A)**. The relative expression levels of gene and protein at 6 h, 12 h, or 24 h post-treatment with 1 ng/mL IL-1 β **(C)**. The relative expression levels of miR-455-3p) and HOTTIP **(A,C)** were assessed by RT-qPCR; While the expression levels of CCL3, Col2a1 Aggrecan, MMP3 and MMP13 **(A–D)** were estimated by RT-qPCR and western blotting. U6, GAPDH and β-actin were used as endogenous controls. The data shown represent the mean ± SD of at least three independent experiments. ^*^p < 0.05, ^∗∗^*p* < 0.01, ^∗∗∗^*p* < 0.001.

Then we confirmed the regulation of miR-455-3p, HOTTIP and CCL3 expression by IL-1β, a well-documented, proinflammatory cytokine found in OA. In normal chondrocytes (taken from two males and two females; age range: 55–65 years), stimulation for 24 h with IL-1β in a dose- and time-dependent manner resulted in decreased miR-455-3p expression and increased HOTTIP and CCL3 expression (Figures 5A,C). IL-1β stimulation led to increased expression of the CCL3 protein in a dose- and time-dependent manner (Figures 5B,D). These results revealed that reduced miR-455-3p expression in response to IL-1β correlate with increased HOTTIP and CCL3 expression in PHCs.

### Negative Regulation of HOTTIP and CCL3 Expression by miR-455-3p in PHCs

To assess whether IL-1β-responsive-HOTTIP and -miR-455-3p regulate CCL3 expression, PHCs were transfected with Control, pcDNA3.1-HOTTIP, pcDNA3.1-HOTTIP + miR-455-3p, siHOTTIP, or siHOTTIP + anti-miR-455-3p; 48 h later, they were stimulated with IL-1β for 12 and 24 h, respectively. Overexpression of HOTTIP in PHCs increased the IL-1β-induced expression of CCL3, MMP3, and MMP13 mRNA, while the effect was partially abolished when miR-455-3p was co-transfected. Conversely, siHOTTIP led to a significant decrease in CCL3 mRNA expression and the effect could be partially restored when anti-miR-455-3p was co-transfected (Supplementary Figure 1). These results suggest that HOTTIP and miR-455-3p competitively regulate CCL3 expression in PHCs.

### HOTTIP Acts as a miR-455-3p Sponge, Enhancing the Expression of CCL3

**FIGURE 6:**
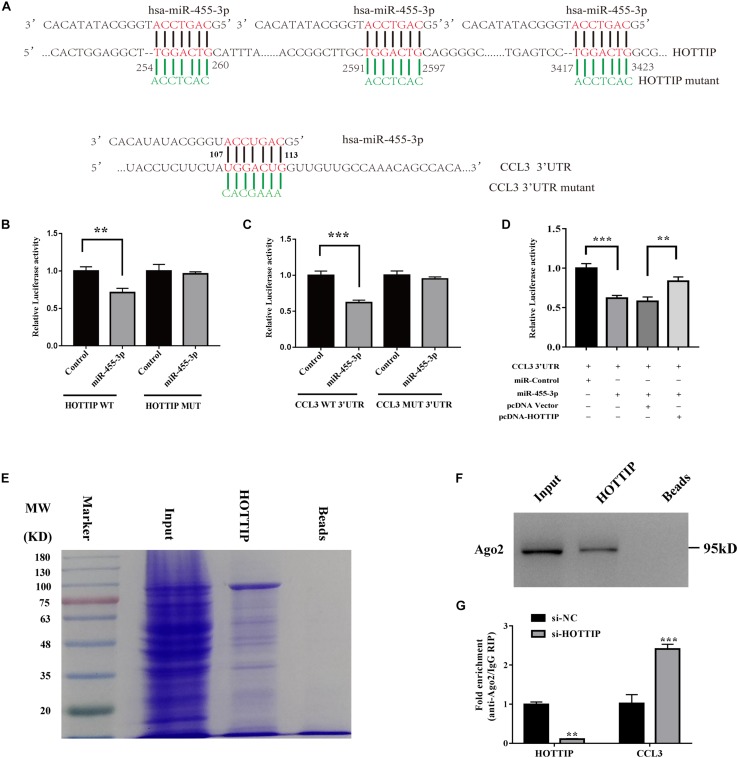
HOTTIP may regulate chondrogenesis and degeneration through ceRNA mechanism *in vitro*. Putative binding site of miR-455-3p in HOTTIP and CCL3 3′-UTR were shown **(A)**. A luciferase reporter carrying the Luc-HOTTIP-wt or Luc-HOTTIP-mut **(B)** or Luc-CCL3-UTR-wt or Luc-CCL3-UTR-mut **(C)** in which the binding site of miR-455-3p was introduced into 293T cells along with negative miR-control (NC), miR-455-3p. The cells were harvested 48 h later for luciferase assays **(B,C)**. CCL3 3′-UTR and miR-455-3p constructs were cotransfected into HEK293 cells with plasmids expressing HOTTIP or with a control vector to verify the ceRNA activity of HOTTIP. Histogram indicates the values of luciferase measured 48 h after transfection **(D)**. HOTTIP was transcribed *in vitro* for RNA pulldown, and then the resolved by in-gradient gel electrophoresis was used for mass spectrometry analysis. These showed a possible interaction exists between HOTTIP and AGO2. Beads were used as control. MW, molecular weight marker (kDal) **(E)**. The binding of HOTTIP and AGO2 was examined by western blot **(F)**. RIP assay of the enrichment of AGO2 on HOTTIP and CCL3 transcripts relative to IgG in PHCs transfected with siHOTTIP **(G)**. The data shown represent the mean ± SD of three independent experiments. ^∗∗^*p* < 0.01, ^∗∗∗^*p* < 0.001.

To further investigate the regulatory relationship of HOTTIP, miR-455-3p, and CCL3, we performed a dual luciferase reporter assay on HOTTIP and CCL3 plasmids. The results showed that luciferase activity was reduced for HOTTIP (Figure 6B) and CCL3 (Figure 6C) as compared with that of the vector control when miR-455-3p was expressed. This data confirms that miR-455-3p can bind directly to both HOTTIP and CCL3 through distinct hsa-miR-455-3p binding sites (Figure 6A). When co-transfected with HOTTIP, luciferase activity of CCL3 was reserved, confirming that HOTTIP can compete with CCL3 in binding to miR-455-3p (Figure 6D). This suggests that HOTTIP acts as an endogenous RNA by binding miR-455-3p, thus eliminating the miRNA-induced suppression of CCL3. Together, this indicates that by binding miR-455-3p, HOTTIP acts as ceRNA to enhance CCL3 mRNA levels. Moreover, to confirm whether HOTTIP is present in miRNA-containing ribonucleoprotein complexes (miRNPs), RNA pull-down assay, MS, and western blot assays were performed, revealing a possible interaction between HOTTIP and AGO2 (Figures 6E,F and Supplementary Table 2). Moreover, a RIP assay was done on AGO2, the vital component of the RNA-induced silencing complex (RISC). As shown in Figure 6G. HOTTIP knockdown contributed to the decreased enrichment of AGO2 in HOTTIP, but substantially increased AGO2 in CCL3 transcripts. Taken together, these results suggest that HOTTIP competes with CCL3 transcripts for the AGO2-based RISC.

### miR-455-3p Deletion Enhances HOTTIP Expression and Accelerates Cartilage Degeneration *in vivo*

**FIGURE 7:**
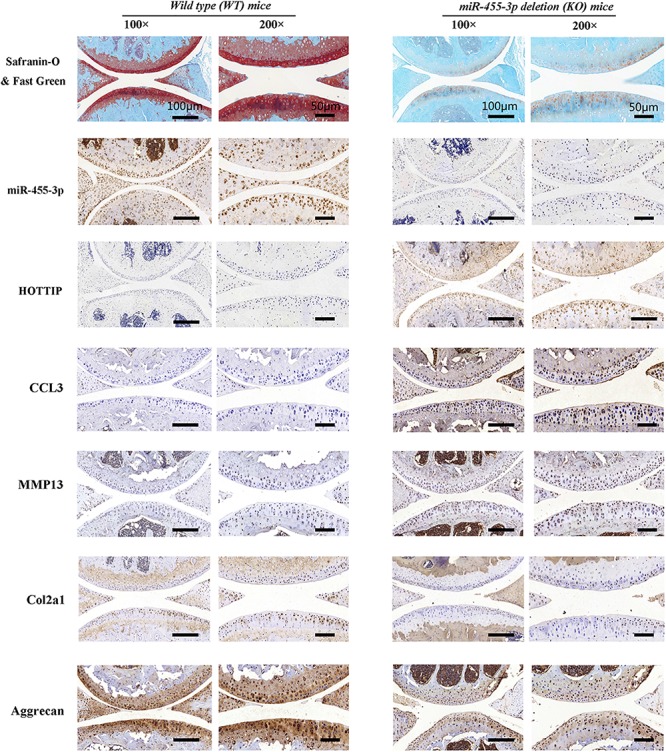
miR-455-3p deletion (KO) mice accelerated cartilage degeneration. Safranin O & Fast green staining, immunohistochemistry (col2a1, aggrecan, MMP13, and CCL3) and *in situ* hybridization (miR-455-3p and HOTTIP) analysis of the knee joint in 12-month-old mice. miR-455-3p deletion enhanced the expression of HOTTIP and CCL3 and accelerated a cartilage degeneration. Data shown are representative of results from five wild-type mice or miR-455-3p-deficient (KO) mice. Scale bar: 50 μm.

To examine the role of miR-455-3p in cartilage homeostasis, we used a miR-455-3p-deficient mouse model. We harvested the knee joints of 10-month-old miR-455-3p^–/–^ and WT mice. miR-455-3p^–/–^ mice showed severe cartilage matrix defects when compared with articular cartilage from WT mice, exhibiting enhanced expression of HOTTIP, CCL3, and MMP13 (Figure 7). Moreover, expression of cartilage-specific genes, such as COL2A1 and aggrecan, was significantly reduced. This supports the hypothesis that miR-455-3p play a critical role in cartilage homeostasis, and its loss is conducive to cartilage degradation characteristic of OA.

### miR-455-3p and HOTTIP Regulate the Function of CCL3 *in vivo*

**FIGURE 8:**
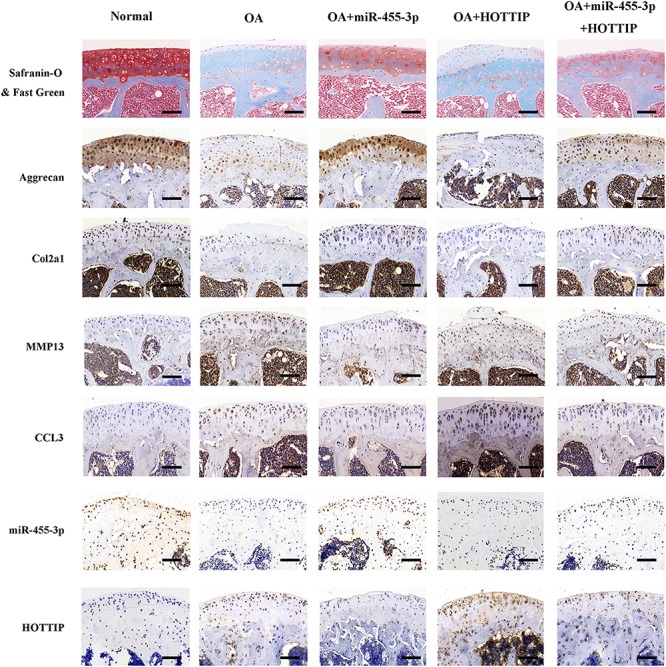
miR-455-3p and lncRNA HOTTIP regulate the function of CCL3 *in vivo*. Sections of tibial plateau (*n* = 10) were stained using Safranin-O/Fast Green immunohistochemistry (COL2A1, aggrecan, MMP13, and CCL3) and *in situ* hybridization (miR-455-3p and HOTTIP probe). Scale bar: 50 μm.

To verify the critical biological functions of these genes *in vivo*, we used collagenase VII-induced OA mice given treatments of agomir-455-3p, pcDNA3.1-HOTTIP, pcDNA3.1-HOTTIP + agomir-455-3p or vehicle (OA) compared against a normal, non-collagenase VII group. Post-treatment, the mice were sacrificed and the knees were harvested to assess the condition of cartilage degradation by *in situ* hybridization, immunohistochemistry, and histology staining. We found that in the HOTTIP overexpression group there was significantly more cartilage degradation that than that of the normal and OA groups. Groups treated with miR-455-3p exhibited diminished cartilage destruction caused by HOTTIP overexpression alone (Figure 8). Thus, consistent with our *in vitro* findings, we discovered that the effect of HOTTIP in promoting cartilage degradation was ameliorated by miR-455-3p in an OA mouse model.

## Discussion

Many researches of OA have focused on the epigenetic regulation of its pathogenesis and potential therapeutic targets, specifically non-coding RNA, including miRNAs and lncRNAs (Barter and Young, 2013). Many reports suggest that miRNAs have gained increasing interest owing to their important regulatory functions in bone and cartilage (Meng et al., 2016; Mao et al., 2017). We demonstrated previously that miR-455-3p expression is upregulated during early chondrogenesis (Zhang et al., 2012, 2015; Chen et al., 2016; Sun H. et al., 2018), revealing that miR-455-3p enhances cartilage development by promoting the expression of certain cartilage-specific genes. However, the role of miR-455-3p in cartilage development and degeneration remain unclear. Previous studies report that lncRNAs play critical roles in the development of bone and cartilage tissue (Hassan et al., 2015; Huynh et al., 2017), suggesting that lncRNAs may influence the balance between anabolic and catabolic processes in joint cartilage and could be applied to diagnosis and prognosis, serving as a personalized therapeutic biomarker to impede, stop, and even reverse OA progression (Steck et al., 2012; Fu et al., 2015). LncRNAs not only regulate the fate of chondrocytes, but also that of arthritis-associated factors. For example, Growth Arrest-Specific 5 (GAS5) suppressed miR-21 expression to inhibit the autophagic response and stimulate apoptosis in OA chondrocytes (Song et al., 2014). Furthermore, HOTTIP may serve as a promotor of cartilage degradation via the inhibition of the HOXA13/integrin-α1 signaling pathway (Kim et al., 2013). According the bioinformatics analysis, the potential involvement of miR-455-3p and HOTTIP has been established. However, the regulatory mechanisms between HOTTIP and miR-455-3p in chondrogenesis and chondrocyte metabolism remain largely unknown.

In this study, we demonstrated for the first time that HOTTIP promotes CCL3 expression in chondrogenesis and cartilage degradation by sponging miR-455-3p. Our data showed that HOTTIP, CCL3, and miR-455-3p were expressed *in vitro*, and elucidated the opposing expression patterns between HOTTIP and miR-455-3p, particularly during the later stages of chondrogenic differentiation. Overexpression HOTTIP promoted the COL10A1, RUNX2, CCL3, MMP3, and MMP13 expression and suppressed chondrogenesis during hMSCs induced to chondrogenesis. However, we found that co-transfected miR-455-3p abolished the biological effect of HOTTIP. In contrast, knockdown of HOTTIP expression dramatically reduced the expression of COL10A1, RUNX2, CCL3, MMP3, and MMP13. As expected, inhibition of miR-455-3p also decreased expression of these genes. Together, these results indicate that HOTTIP inhibited chondrogenic differentiation of hMSCs by enhancing CCL3 expression and absorbing miR-455-3p, thereby reducing cartilage-specific gene expression and leading to cartilage degradation.

Moreover, we also verified that the expression levels of HOTTIP and CCL3 are significantly upregulated while that of miR-455-3p is downregulated in OA tissues. Proinflammatory cytokines such as IL-1β (Goldring and Otero, 2011) and anti-inflammatory therapies represent innovative approaches in OA (Calich et al., 2010). In this study, we found that miR-455-3p was downregulated in a time (6, 12, and 24 h)- and dose (0, 0.01, 0.1, and 1 ng/mL)-dependent manner in chondrocytes stimulated with IL-1β. Overexpression of miR-455-3p inhibits ADAMTS-4, ADAMTS-5, MMP3, and MMP13 expression in chondrocytes and increases expression of cartilage-specific genes such as aggrecan, COL2A1, and SOX9, by targeting HOTTIP and CCL3 in PHCs. We further confirmed that miR-455-3p deletion resulted in loss of the cartilage matrix and reduction of cartilage-specific gene expression, such as that of COL2A1 and aggrecan in miR-455-3p-deficient mice. Moreover, miR-455-3p deletion enhanced HOTTIP and CCL3 expression when compared to WT mice. However, HOTTIP was upregulated in a time- and dose-dependent manner in chondrocytes stimulated with IL-1β. Overexpression of HOTTIP promotes CCL3, MMP3, and MMP13 expression and reduces the expression of COL2A1 and aggrecan, the effects of which are abolished by co-transfection with miR-455-3p in PHCs. Gain- and loss-of-function assays of CCL3 with PHCs and detected the expression of cartilage-specific gene and protein. The results suggested that CCL3 may aggravate cartilage degradation.

According the bioinformatic analysis revealed that miR-455-3p was predicted to form complementary base pairing with HOTTIP and chemotactic factor CCL3. Using a luciferase reporter assay, we confirmed that miR-455-3p could bind directly to HOTTIP (nucleotide position 254-260, 2591-2597, 3417-3423, and CCL3 (nucleotide position 107-113) simultaneously. Then we use the Luciferase reporter assay to indicate that miR-455-3p probably interacts with HOTTIP and CCL3 and downregulates their expression. Co-transfection luciferase reporter assay revealed that HOTTIP reversed the inhibited reporter plasmid luciferase activity for CCL3, which confirmed that HOTTIP can compete with CCL3 by sponging miR-455-3p. Then we did the RNA pull-down assay, western blot and MS analysis. We found that HOTTIP was present in miRNPs and may interact with Ago2 protein directly, which is consistent with previous studies (Sun et al., 2017; Sun Y. et al., 2018; Zheng et al., 2019). We also find other RNPs, such as RS5, RS21, RS23, RL5, RT34, RT14, and RM48, were showed in the Supplementary Table 2. Furthermore, HOTTIP and miR-455-3p co-immunoprecipitation with anti-AGO2 showed a positive physical interaction in PHCs, which supports HOTTIP’s role as a miRNA sponge. Inverse expression correlation between HOTTIP and miR-455-3p in OA and normal cartilage tissues, verified that HOTTIP acts as endogenous sponge for miR-455-3p. Decreased ectopic expression of miR-455-3p^–/–^ promoted cartilage matrix loss and destruction, which was consistent with the effects of HOTTIP overexpression *in vivo*. Taken together, our data reveal for the first time that HOTTIP functions as ceRNA by competing with miR-455-3p in the regulation of CCL3 expression in chondrogenesis and cartilage degradation. Consistent with our *in vitro* findings, we further found that the effect of HOTTIP on the acceleration of OA progression can be ameliorated by miR-455-3p in a collagenase VII-induced OA mouse model.

## Conclusion

We elucidated a ceRNA regulatory network of HOTTIP/miR-455-3p/CCL3 in the chondrogenesis and pathogenesis of OA. These results reveal a possible mechanism for HOTTIP as a crucial regulator in OA pathogenesis owing to its ability to suppress miR-455-3p in cartilage degradation. We propose that the targeting of HOTTIP may help in the development of novel therapeutics to combat OA pathogenesis.

## Data Availability

The datasets generated for this study can be found in the PRIDE archive, accession PXD014988 https://www.ebi.ac.uk/pride/archive/projects/PXD014988.

## Ethics Statement

The studies involving human participants were reviewed and approved by The Ethics Committee of the First Affiliated Hospital of Sun Yat-sen University. The patients/participants provided their written informed consent to participate in this study. The animal study was reviewed and approved by The Animal Research Committee of the First Affiliated Hospital of Sun Yat-sen University.

## Author Contributions

WL and ZhZ conceived and designed the study. GM, YK, SH, RL, and ZiZ acquired, analyzed, and interpreted the data. GM drafted and edited the manuscript. GM, YK, SH, RL, and ZhZ critically revised the manuscript for important intellectual content. All authors approved the final version of the manuscript.

## Conflict of Interest Statement

The authors declare that the research was conducted in the absence of any commercial or financial relationships that could be construed as a potential conflict of interest.

## Footnotes

1 https://cm.jefferson.edu/
