# Supplementary material for: Long Non-coding RNA HOTTIP Promotes CCL3 Expression and Induces Cartilage Degradation by Sponging miR-455-3p
Source: Front Cell Dev Biol. 2019 Aug 23;7:161. doi: 10.3389/fcell.2019.00161 (PMC6716540; doi:10.3389/fcell.2019.00161)
Supplement: Supplementary file 1 [file Table_1.DOCX]

Supplementary table1.Primers for quantitative real-time polymerase chain reaction (qRT-PCR)

| Gene |  | primer sequence(5'-3') |
| --- | --- | --- |
| hsa-COL2A1 | F | GCACCTGCAGAGACCTGAAAC |
| hsa-COL2A1 | R | GCAAGTCTCGCCAGTCTCCA |
| hsa-HOTTIP | F | AACGATGTGTGTGTGCCTTGAT |
| hsa-HOTTIP | R | TGGTCCGACAGGGTGAATT |
| hsa-COL10A1 | F | CATAAAAGGCCCACTACCCAAC |
| hsa-COL10A1 | R | ACCTTGCTCTCCTCTTACTGC |
| hsa-SOX9 | F | GGAGATGAAATCTGTTCTGGGAATG |
| hsa-SOX9 | R | TTGAAGGTTAACTGCTGGTGTTCTG |
| hsa-RUNX2 | F | CACTGGCGCTGCAACAAGA |
| hsa-RUNX2 | R | CATTCCGGAGCTCAGCAGAATAA |
| hsa-CCL3 | F | AGTTCTCTGCATCACTTGCTG |
| hsa-CCL3 | R | CGGCTTCGCTTGGTTAGGAA |
| hsa-COMP | F | GATCACGTTCCTGAAAAACACG |
| hsa-COMP | R | GCTCTCCGTCTGGATGCAG |
| hsa-Aggrecan | F | GATGTTCCCTGCAATTACCACCTC |
| hsa-Aggrecan | R | TGATCTCATACCGGTCCTTCTTCTG |
| hsa-MMP-13 | F | TCCTGATGTGGGTGAATACAATG |
| hsa-MMP-13 | R | GCCATCGTGAAGTCTGGTAAAAT |
| hsa-MMP-3 | F | CGGTTCCGCCTGTCTCAAG |
| hsa-MMP-3 | R | CGCCAAAAGTGCCTGTCTT |
| hsa-ADAMTS4 | F | GGTCAAGGTCCCATGTGCAAC |
| hsa-ADAMTS4 | R | GAATGCGGCCATCTTGTCATC |
| hsa-ADAMTS5 | F | AATGCACTTCAGCCACCATCA |
| hsa-ADAMTS5 | R | TCGTAGGTCTGTCCTGGGAGTTC |
| hsa-GAPDH | F | GCACCGTCAAGGCTGAGAAC |
| hsa-GAPDH | R | TGGTGAAGACGCCAGTGGA |
| hsa-U6 | F | CTCGCTTCGGCAGCACA |
| hsa-U6 | R | AACGCTTCACGAATTTGCGT |
| hsa-miR-455-3p | F | GCAGTCCATGGGCATATACAC |
