# Supplementary material for: Long Non-coding RNA HOTTIP Promotes CCL3 Expression and Induces Cartilage Degradation by Sponging miR-455-3p
Source: Front Cell Dev Biol. 2019 Aug 23;7:161. doi: 10.3389/fcell.2019.00161 (PMC6716540; doi:10.3389/fcell.2019.00161)
Supplement: Supplementary file 2 [file Table_2.pdf]

Supplementary Table 2 Mass spectrometry analysis of the proteins pulled down by HOTTIP

Hits	Protein Name	Score	Mass	Matches	Sequences	emPAI
1	TBB2A_HUMAN	1290	50274	82 (58)	19 (15)	3.04
2	NUCL_HUMAN	1162	76625	75 (49)	26 (17)	1.22
3	ACTG_HUMAN	1150	42108	57 (47)	18 (15)	5.14
4	TBA1A_HUMAN	908	50788	50 (42)	18 (16)	3.81
5	ROA2_HUMAN	701	37464	35 (31)	13 (11)	3.99
6	SYEP_HUMAN	701	172080	54 (33)	40 (25)	0.69
7	H14_HUMAN	598	21852	33 (25)	12 (10)	4.54
8	ROA3_HUMAN	585	39799	29 (24)	11 (10)	2.31
9	F120A_HUMAN	575	123008	26 (21)	19 (17)	0.65
10	ILF3_HUMAN	534	95678	30 (21)	20 (15)	1.03
11	HNRPR_HUMAN	513	71184	34 (23)	18 (11)	1.15
12	HNRPQ_HUMAN	491	69788	37 (22)	19 (12)	1.09
13	PABP1_HUMAN	490	70854	34 (22)	21 (14)	1.16
14	GT251_HUMAN	465	71933	36 (24)	21 (16)	1.04
15	SYRC_HUMAN	452	76129	37 (26)	22 (20)	1.75
16	FUBP1_HUMAN	449	67690	27 (16)	21 (12)	1.04
17	AP2A2_HUMAN	447	104807	34 (19)	23 (15)	0.69
18	XRCC5_HUMAN	408	83222	35 (20)	21 (13)	0.86
19	HNRPL_HUMAN	396	64720	22 (15)	13 (11)	1
20	SRSF2_HUMAN	378	25461	11 (8)	6 (3)	0.85
21	CN37_HUMAN	376	47948	27 (15)	18 (10)	1.54
22	ROA1_HUMAN	358	38837	31 (22)	11 (7)	1.46
23	DDX58_HUMAN	337	108014	30 (16)	21 (14)	0.56
24	HNRH1_HUMAN	336	49484	15 (10)	9 (5)	0.57
25	HNRH2_HUMAN	329	49517	15 (9)	9 (4)	0.47
26	SRSF1_HUMAN	316	27842	19 (14)	11 (10)	2.46
27	SEC63_HUMAN	297	88341	20 (15)	17 (13)	0.73
28	EIFCL_HUMAN	282	106091	21 (11)	16 (11)	0.4
29	RECQ1_HUMAN	280	74436	16 (10)	12 (8)	0.47
30	AP3B1_HUMAN	280	121815	31 (16)	26 (15)	0.49
31	SND1_HUMAN	273	102618	25 (13)	21 (12)	0.5
32	SFPQ_HUMAN	265	76216	26 (16)	18 (10)	0.66
33	ZCCHV_HUMAN	253	103135	19 (11)	14 (7)	0.37
34	K1C17_HUMAN	252	48361	14 (9)	14 (9)	0.81
35	HNRPM_HUMAN	252	77749	21 (11)	15 (7)	0.51
36	SRSF6_HUMAN	247	39677	24 (15)	15 (11)	1.42
37	H2B1C_HUMAN	245	13898	24 (15)	10 (6)	4.8
38	RTCB_HUMAN	243	55688	24 (9)	16 (9)	0.68
39	FUBP2_HUMAN	239	73355	15 (8)	13 (7)	0.42
40	HNRL2_HUMAN	238	85622	20 (9)	16 (9)	0.4
41	DDX5_HUMAN	233	69618	25 (13)	15 (10)	0.66

42	FND3B_HUMAN	231	134970	14 (7)	12 (6)	0.18
43	RBP2_HUMAN	231	362365	97 (5)	21 (4)	0.04
44	YBOX3_HUMAN	226	40066	10 (8)	6 (5)	0.74
45	NONO_HUMAN	225	54311	27 (11)	17 (7)	0.8
46	LA_HUMAN	224	46979	14 (10)	12 (8)	0.84
47	TXLNA_HUMAN	219	62195	19 (10)	14 (10)	0.67
48	ILF2_HUMAN	216	43263	11 (9)	7 (6)	0.67
49	HMGB3_HUMAN	211	23137	14 (10)	4 (2)	0.31
50	RAB10_HUMAN	211	22755	14 (8)	10 (5)	1.28
51	SYIC_HUMAN	200	145718	19 (8)	14 (7)	0.19
52	KINH_HUMAN	199	110358	21 (7)	18 (7)	0.23
53	PABP4_HUMAN	197	71080	13 (8)	10 (6)	0.37
54	PRKRA_HUMAN	188	34839	11 (7)	10 (6)	0.89
55	BGH3_HUMAN	188	75261	15 (10)	13 (8)	0.47
56	NMT1_HUMAN	187	57112	9 (5)	8 (4)	0.32
57	RHOA_HUMAN	186	22096	7 (6)	7 (6)	1.33
58	U2AF1_HUMAN	182	28368	11 (8)	7 (5)	0.94
59	CO6A3_HUMAN	182	345167	55 (10)	30 (10)	0.1
60	SYMC_HUMAN	178	102249	24 (10)	15 (8)	0.37
61	MYH10_HUMAN	175	229827	22 (6)	17 (6)	0.09
62	H2A1D_HUMAN	173	14099	16 (9)	5 (3)	1.38
63	PCBP3_HUMAN	168	39725	7 (5)	5 (4)	0.38
64	ABCF1_HUMAN	161	96323	11 (3)	9 (3)	0.11
65	STAU1_HUMAN	158	63428	8 (6)	7 (5)	0.35
66	DDX21_HUMAN	158	87804	16 (7)	13 (7)	0.29
67	SRSF4_HUMAN	157	56759	13 (9)	9 (7)	0.48
68	ROA0_HUMAN	156	30993	10 (8)	6 (4)	0.5
69	HNRL1_HUMAN	153	96250	16 (6)	13 (6)	0.22
70	RPN2_HUMAN	152	69355	12 (8)	9 (6)	0.45
71	FINC_HUMAN	151	266052	14 (5)	14 (5)	0.06
72	SRRT_HUMAN	150	101060	17 (9)	16 (9)	0.33
73	RUVB2_HUMAN	150	51296	11 (6)	9 (5)	0.45
74	RL1D1_HUMAN	149	55167	14 (9)	13 (9)	0.68
75	SGPL1_HUMAN	148	64053	15 (5)	13 (5)	0.28
76	1C03_HUMAN	147	41234	8 (3)	6 (3)	0.26
77	PACN3_HUMAN	144	48799	5 (2)	3 (1)	0.14
78	IFM1_HUMAN	141	14126	3 (2)	2 (1)	0.54
79	STT3B_HUMAN	137	94241	19 (8)	12 (6)	0.23
80	SEPT7_HUMAN	136	50933	11 (7)	7 (3)	0.37
81	TGM2_HUMAN	134	78420	11 (5)	10 (5)	0.23
82	THUM1_HUMAN	134	39690	8 (5)	8 (5)	0.49
83	RHOC_HUMAN	133	22334	6 (6)	6 (6)	1.32
84	ROAA_HUMAN	132	36316	7 (5)	4 (3)	0.3
85	SR140_HUMAN	132	118675	20 (6)	14 (6)	0.18

86	MYO1E_HUMAN	132	127552	13 (4)	11 (4)	0.11
87	GFPT2_HUMAN	130	77680	18 (8)	11 (6)	0.28
88	EMC2_HUMAN	130	34982	2 (2)	2 (2)	0.2
89	SYYC_HUMAN	130	59448	13 (7)	13 (7)	0.46
90	SRSF5_HUMAN	128	31359	7 (4)	4 (2)	0.35
91	SYSC_HUMAN	128	59253	11 (3)	11 (3)	0.18
92	VAPA_HUMAN	128	28103	9 (6)	6 (4)	0.75
93	H2A1B_HUMAN	127	14127	15 (8)	5 (3)	1.38
94	MCA3_HUMAN	125	19855	9 (5)	9 (5)	1.19
95	DDX46_HUMAN	125	117803	14 (5)	12 (4)	0.15
96	HNRDL_HUMAN	123	46580	10 (5)	7 (3)	0.23
97	ABCE1_HUMAN	123	68240	11 (6)	11 (6)	0.33
98	TRI25_HUMAN	123	72581	9 (4)	9 (4)	0.19
99	PAPS2_HUMAN	120	70027	12 (6)	10 (6)	0.32
100	FUBP3_HUMAN	119	61944	15 (5)	10 (3)	0.23
101	SRSF7_HUMAN	119	27578	10 (4)	7 (4)	0.58
102	RAB12_HUMAN	118	27573	9 (4)	6 (2)	0.26
103	PCOC1_HUMAN	118	48797	6 (5)	5 (5)	0.39
104	MATR3_HUMAN	115	95078	12 (8)	7 (5)	0.31
105	OAS2_HUMAN	115	83348	5 (4)	5 (4)	0.17
106	CHM4B_HUMAN	114	24935	5 (2)	4 (2)	0.29
107	AP3S1_HUMAN	114	21946	4 (2)	3 (2)	0.33
108	ATD3A_HUMAN	113	71610	18 (6)	15 (6)	0.31
109	CO5A2_HUMAN	112	145790	18 (7)	13 (6)	0.17
110	PPIP1_HUMAN	111	48018	3 (2)	3 (2)	0.14
111	LARP1_HUMAN	110	123833	14 (4)	12 (4)	0.11
112	RAPH1_HUMAN	109	135799	9 (5)	8 (5)	0.13
113	TRPV4_HUMAN	108	98960	8 (3)	7 (3)	0.1
114	IF2P_HUMAN	108	139198	17 (4)	15 (4)	0.1
115	GSLG1_HUMAN	106	138341	7 (3)	7 (3)	0.07
116	RS5_HUMAN	106	23033	11 (5)	6 (4)	0.97
117	COBA2_HUMAN	106	172484	14 (6)	9 (5)	0.1
118	DDX3X_HUMAN	101	73597	14 (7)	11 (5)	0.3
119	SRP68_HUMAN	100	71199	8 (4)	6 (4)	0.2
120	FUS_HUMAN	100	53622	7 (3)	5 (2)	0.2
121	AIMP2_HUMAN	99	35668	8 (4)	7 (4)	0.43
122	SMC1A_HUMAN	98	143771	24 (7)	16 (6)	0.14
123	HNRPC_HUMAN	97	33707	15 (5)	9 (4)	0.46
124	TR150_HUMAN	97	108658	15 (5)	12 (4)	0.16
125	CO8A1_HUMAN	97	73431	6 (2)	3 (1)	0.09
126	CCPG1_HUMAN	96	88027	11 (3)	10 (2)	0.12
127	PPIG_HUMAN	96	89077	7 (3)	4 (2)	0.11
128	SAMD9_HUMAN	96	185704	17 (2)	11 (2)	0.04
129	RBMS1_HUMAN	96	44705	5 (3)	5 (3)	0.24

130	RUVB1_HUMAN	95	50538	6 (1)	5 (1)	0.07
131	DDX54_HUMAN	95	98819	11 (3)	9 (3)	0.1
132	DDX6_HUMAN	95	54781	13 (6)	11 (6)	0.42
133	DRG1_HUMAN	94	40802	6 (2)	6 (2)	0.17
134	SYLC_HUMAN	94	135577	15 (4)	14 (3)	0.07
135	IQEC1_HUMAN	91	109103	8 (4)	8 (4)	0.13
136	RBM25_HUMAN	91	100467	4 (3)	2 (2)	0.1
137	PSPC1_HUMAN	89	58820	11 (2)	10 (2)	0.11
138	FA98B_HUMAN	89	37566	4 (1)	4 (1)	0.09
139	WASF2_HUMAN	89	54478	7 (3)	5 (2)	0.19
140	EIF3A_HUMAN	88	166867	17 (3)	16 (3)	0.06
141	TOP2B_HUMAN	88	184122	12 (4)	12 (4)	0.07
142	CEMIP_HUMAN	88	154440	14 (3)	11 (3)	0.06
143	MRCKB_HUMAN	88	196189	17 (3)	15 (3)	0.05
144	M4K5_HUMAN	86	96063	28 (2)	10 (2)	0.07
145	DRG2_HUMAN	86	41120	6 (3)	5 (2)	0.17
146	PNKP_HUMAN	86	57554	8 (4)	7 (4)	0.25
147	RAB5A_HUMAN	85	23872	4 (3)	4 (3)	0.48
148	CPSF7_HUMAN	85	52189	5 (3)	4 (3)	0.2
149	DNJA2_HUMAN	85	46344	7 (2)	7 (2)	0.15
150	DHB4_HUMAN	85	80092	8 (1)	6 (1)	0.04
151	ERLN1_HUMAN	83	39072	2 (2)	2 (2)	0.18
152	SELB_HUMAN	83	65890	8 (5)	5 (4)	0.28
153	IDUA_HUMAN	83	72967	8 (4)	7 (3)	0.14
154	SAMH1_HUMAN	82	72896	3 (2)	3 (2)	0.09
155	CATB_HUMAN	82	38766	4 (4)	4 (4)	0.39
156	FBRL_HUMAN	82	33877	12 (5)	10 (4)	0.45
157	EIF3I_HUMAN	81	36878	5 (2)	5 (2)	0.19
158	RAB21_HUMAN	81	24731	8 (3)	7 (3)	0.46
159	GLYM_HUMAN	80	56414	14 (3)	11 (3)	0.19
160	DAZP1_HUMAN	80	43584	5 (2)	4 (2)	0.16
161	TPSN_HUMAN	79	47938	6 (2)	6 (2)	0.14
162	EIF3F_HUMAN	79	37654	4 (3)	4 (3)	0.29
163	CPNE2_HUMAN	78	61835	7 (4)	6 (3)	0.17
164	PGAM5_HUMAN	78	32213	5 (2)	3 (2)	0.22
165	B3GA3_HUMAN	78	37270	6 (2)	5 (2)	0.19
166	FXR1_HUMAN	78	70020	8 (4)	7 (3)	0.2
167	TIAR_HUMAN	78	41906	7 (2)	7 (2)	0.16
168	U2AF2_HUMAN	77	53809	4 (2)	3 (1)	0.06
169	G3BP1_HUMAN	77	52189	4 (1)	4 (1)	0.06
170	SSRP1_HUMAN	77	81367	8 (4)	7 (4)	0.17
171	JUNB_HUMAN	76	36028	3 (1)	3 (1)	0.09
172	HELZ2_HUMAN	76	298286	26 (3)	16 (3)	0.03
173	NIBL1_HUMAN	75	84598	8 (3)	8 (3)	0.12

174	SF3B3_HUMAN	75	136575	11 (3)	8 (3)	0.07
175	HNRLL_HUMAN	74	60900	5 (2)	4 (2)	0.11
176	SC11A_HUMAN	74	20612	4 (3)	4 (3)	0.57
177	EIF3E_HUMAN	74	52587	5 (2)	5 (2)	0.13
178	GTF2I_HUMAN	73	112859	14 (4)	11 (4)	0.12
179	ODO1_HUMAN	72	117059	9 (4)	9 (4)	0.12
180	MLEC_HUMAN	71	32385	3 (2)	3 (2)	0.22
181	PHF5A_HUMAN	71	13138	2 (1)	2 (1)	0.26
182	RFA1_HUMAN	70	68723	5 (1)	5 (1)	0.05
183	ZN207_HUMAN	70	51002	1 (1)	1 (1)	0.06
184	RAP1B_HUMAN	69	21040	5 (3)	5 (3)	0.56
185	ACD11_HUMAN	69	88026	5 (1)	5 (1)	0.04
186	HERC5_HUMAN	69	118203	13 (4)	13 (4)	0.12
187	TENS1_HUMAN	69	186499	8 (1)	7 (1)	0.02
188	SYFB_HUMAN	69	66701	9 (6)	7 (5)	0.33
189	PTH2_HUMAN	68	19466	7 (2)	5 (1)	0.38
190	IF5_HUMAN	68	49648	11 (2)	9 (2)	0.14
191	PRP19_HUMAN	67	55603	4 (4)	4 (4)	0.26
192	PHB_HUMAN	67	29843	3 (2)	3 (2)	0.24
193	RHOG_HUMAN	67	21751	4 (1)	4 (1)	0.15
194	MAGT1_HUMAN	66	38411	9 (4)	7 (4)	0.39
195	DENR_HUMAN	66	22477	3 (2)	3 (2)	0.32
196	RCC2_HUMAN	66	56790	7 (5)	6 (4)	0.25
197	SC22B_HUMAN	66	24806	4 (2)	4 (2)	0.29
198	CRIP2_HUMAN	66	23276	1 (1)	1 (1)	0.14
199	NSUN2_HUMAN	65	87214	16 (3)	12 (3)	0.12
200	CPNE6_HUMAN	64	62864	4 (4)	3 (3)	0.17
201	EHD1_HUMAN	64	60646	6 (2)	5 (2)	0.11
202	TPM3_HUMAN	64	32987	8 (1)	5 (1)	0.1
203	H31T_HUMAN	64	15613	7 (4)	5 (3)	0.8
204	RPAB3_HUMAN	64	17189	5 (1)	4 (1)	0.2
205	TSR1_HUMAN	64	92151	12 (3)	9 (3)	0.11
206	SPT5H_HUMAN	64	121324	11 (3)	10 (3)	0.08
207	PURA_HUMAN	64	35003	5 (3)	4 (2)	0.2
208	BAP31_HUMAN	63	28031	4 (2)	4 (2)	0.25
209	KC1A_HUMAN	63	39118	11 (3)	8 (3)	0.28
210	AGO3_HUMAN	63	98495	10 (2)	8 (2)	0.07
211	IF2B2_HUMAN	63	66195	5 (2)	4 (2)	0.1
212	RALY_HUMAN	62	32501	6 (2)	4 (2)	0.21
213	SYFA_HUMAN	62	57585	8 (3)	6 (3)	0.18
214	VP33B_HUMAN	62	71225	3 (2)	3 (2)	0.09
215	TMM43_HUMAN	62	44904	5 (3)	4 (2)	0.24
216	CATZ_HUMAN	61	34530	1 (1)	1 (1)	0.1
217	AKP8L_HUMAN	61	72060	6 (1)	5 (1)	0.05

218	PLCD3_HUMAN	61	90115	8 (2)	7 (2)	0.07
219	IFIT5_HUMAN	60	56268	13 (4)	8 (3)	0.19
220	SMCE1_HUMAN	60	46678	4 (1)	4 (1)	0.07
221	POGZ_HUMAN	60	157355	6 (1)	6 (1)	0.02
222	THOC4_HUMAN	60	26872	4 (2)	3 (1)	0.26
223	FA98A_HUMAN	60	55823	5 (1)	5 (1)	0.06
224	PARP9_HUMAN	60	97308	6 (2)	4 (2)	0.07
225	ZFR_HUMAN	60	118079	19 (1)	6 (1)	0.03
226	CO5A1_HUMAN	59	184131	16 (4)	13 (4)	0.07
227	RS21_HUMAN	59	9220	3 (1)	2 (1)	0.38
228	ACL6A_HUMAN	59	47944	3 (2)	3 (2)	0.14
229	SYCC_HUMAN	59	86103	7 (3)	7 (3)	0.12
230	DHX15_HUMAN	58	91673	8 (4)	8 (4)	0.15
231	LARP7_HUMAN	58	67143	9 (1)	7 (1)	0.05
232	EIF3K_HUMAN	58	25329	3 (1)	2 (1)	0.13
233	PAR12_HUMAN	58	80496	5 (2)	3 (2)	0.08
234	CPSF5_HUMAN	57	26268	2 (2)	2 (2)	0.27
235	MCM3_HUMAN	57	91551	7 (3)	7 (3)	0.11
236	SYHM_HUMAN	57	57593	10 (3)	8 (3)	0.18
237	STT3A_HUMAN	56	81104	16 (3)	8 (2)	0.08
238	CSDE1_HUMAN	56	89684	14 (2)	9 (2)	0.07
239	DPM1_HUMAN	56	29673	3 (1)	3 (1)	0.11
240	HERC6_HUMAN	56	116535	6 (1)	6 (1)	0.03
241	DDX23_HUMAN	56	95866	9 (2)	7 (2)	0.07
242	CP1B1_HUMAN	56	61263	6 (1)	6 (1)	0.05
243	SSRG_HUMAN	56	21067	2 (1)	2 (1)	0.16
244	COPE_HUMAN	55	34688	6 (2)	5 (2)	0.2
245	TADBP_HUMAN	55	45053	4 (3)	4 (3)	0.24
246	CRAC1_HUMAN	55	72174	2 (1)	2 (1)	0.05
247	PDLI5_HUMAN	55	65102	3 (1)	3 (1)	0.05
248	DDX24_HUMAN	55	96899	8 (4)	7 (4)	0.14
249	AGO2_HUMAN	55	98400	15(1)	12(1)	0.03
250	AGO1_HUMAN	55	98293	8 (1)	6 (1)	0.03
251	NFIB_HUMAN	54	47754	3 (2)	3 (2)	0.14
252	DSRAD_HUMAN	54	137178	17 (5)	13 (5)	0.12
253	MYOF_HUMAN	54	236100	11 (1)	11 (1)	0.01
254	DTX3L_HUMAN	54	84585	8 (2)	7 (2)	0.08
255	SDA1_HUMAN	54	80277	7 (1)	6 (1)	0.04
256	DKC1_HUMAN	53	58094	10 (3)	8 (2)	0.18
257	RBM39_HUMAN	53	59628	4 (2)	2 (2)	0.11
258	HDAC2_HUMAN	53	55899	5 (1)	4 (1)	0.06
259	DDX60_HUMAN	53	199722	11 (3)	11 (3)	0.05
260	KCD12_HUMAN	53	35964	1 (1)	1 (1)	0.09
261	RPAB1_HUMAN	53	24650	4 (3)	4 (3)	0.46

262	GREM1_HUMAN	53	21197	1 (1)	1 (1)	0.16
263	SP16H_HUMAN	53	120409	7 (2)	7 (2)	0.06
264	SRSF9_HUMAN	52	25640	3 (3)	3 (3)	0.44
265	F263_HUMAN	52	60370	8 (2)	6 (2)	0.11
266	RTCA_HUMAN	52	39825	5 (2)	4 (2)	0.17
267	PSMD4_HUMAN	52	40939	2 (2)	1 (1)	0.17
268	WAC2C_HUMAN	52	144752	9 (1)	7 (1)	0.02
269	GT252_HUMAN	51	73220	7 (2)	6 (2)	0.09
270	CPSF6_HUMAN	51	59344	9 (2)	6 (2)	0.11
271	IF1AX_HUMAN	51	16564	3 (3)	3 (3)	0.75
272	UE2NL_HUMAN	51	17366	4 (1)	3 (1)	0.2
273	DDX1_HUMAN	51	83349	8 (2)	8 (2)	0.08
274	TENX_HUMAN	50	464946	12 (1)	12 (1)	0.01
275	S10A4_HUMAN	50	11949	3 (1)	2 (1)	0.29
276	SNUT1_HUMAN	50	90371	6 (1)	4 (1)	0.04
277	EHD4_HUMAN	50	61365	10 (1)	5 (1)	0.05
278	NC2A_HUMAN	49	22450	3 (2)	3 (2)	0.32
279	SEC13_HUMAN	49	36031	4 (1)	3 (1)	0.09
280	SLFN5_HUMAN	49	102530	6 (2)	5 (2)	0.06
281	DJC10_HUMAN	49	92333	4 (1)	4 (1)	0.04
282	BAIP2_HUMAN	49	61115	4 (1)	4 (1)	0.05
283	PLD3_HUMAN	48	55127	2 (1)	2 (1)	0.06
284	CO6A2_HUMAN	48	109709	13 (2)	13 (2)	0.06
285	DC1I2_HUMAN	48	71811	1 (1)	1 (1)	0.05
286	SRPRA_HUMAN	48	70223	10 (2)	10 (2)	0.1
287	KHDR1_HUMAN	48	48311	4 (1)	4 (1)	0.07
288	IBP7_HUMAN	48	30138	2 (2)	2 (2)	0.23
289	CAZA1_HUMAN	48	33073	2 (1)	2 (1)	0.1
290	XRN2_HUMAN	48	109426	14 (1)	10 (1)	0.03
291	DDX18_HUMAN	48	75702	7 (1)	7 (1)	0.04
292	RAD21_HUMAN	48	71930	2 (1)	2 (1)	0.05
293	SMHD1_HUMAN	48	227942	16 (2)	15 (2)	0.03
294	HPLN4_HUMAN	48	43402	4 (1)	3 (1)	0.08
295	SQSTM_HUMAN	48	48455	2 (1)	2 (1)	0.07
296	PPIA_HUMAN	47	18229	3 (2)	3 (2)	0.41
297	CDC42_HUMAN	47	21587	4 (1)	4 (1)	0.16
298	SART3_HUMAN	47	110721	7 (1)	5 (1)	0.03
299	PDCD6_HUMAN	47	21912	3 (2)	3 (2)	0.33
300	SPCS3_HUMAN	46	20358	1 (1)	1 (1)	0.17
301	RB11B_HUMAN	46	24588	2 (1)	2 (1)	0.14
302	TMCO1_HUMAN	46	21389	2 (1)	2 (1)	0.16
303	SPCS1_HUMAN	46	11854	1 (1)	1 (1)	0.29
304	PA2G4_HUMAN	45	44101	9 (2)	8 (2)	0.16
305	RBMX_HUMAN	45	42306	18 (1)	10 (1)	0.08

306	E2AK2_HUMAN	45	62512	4 (2)	4 (2)	0.11
307	CBLN4_HUMAN	45	21908	3 (2)	2 (1)	0.15
308	MYO6_HUMAN	45	150965	14 (3)	13 (3)	0.07
309	MICU2_HUMAN	45	49919	6 (1)	6 (1)	0.07
310	CTCF_HUMAN	44	84272	4 (1)	3 (1)	0.04
311	ERH_HUMAN	44	12422	5 (3)	4 (3)	1.08
312	RFC3_HUMAN	44	41328	6 (1)	5 (1)	0.08
313	BRD4_HUMAN	44	152580	11 (1)	11 (1)	0.02
314	GNAS1_HUMAN	44	111697	5 (1)	5 (1)	0.03
315	WASC4_HUMAN	44	137343	10 (1)	9 (1)	0.02
316	SF3B2_HUMAN	43	100279	14 (2)	9 (2)	0.07
317	SCYL1_HUMAN	43	90202	4 (3)	4 (3)	0.11
318	SPB1_HUMAN	43	96898	7 (1)	5 (1)	0.03
319	DNJB4_HUMAN	42	38011	4 (1)	3 (1)	0.09
320	ELAV1_HUMAN	42	36240	5 (1)	3 (1)	0.09
321	TSN_HUMAN	42	26281	2 (1)	2 (1)	0.13
322	ASSY_HUMAN	42	46786	4 (2)	3 (2)	0.15
323	ZFY21_HUMAN	42	27229	6 (1)	4 (1)	0.12
324	LANC2_HUMAN	42	51677	3 (1)	3 (1)	0.06
325	NB5R1_HUMAN	42	34244	3 (1)	3 (1)	0.1
326	TIMP3_HUMAN	42	24813	2 (1)	2 (1)	0.13
327	TRM61_HUMAN	42	31704	1 (1)	1 (1)	0.1
328	DIP2B_HUMAN	42	173606	12 (1)	11 (1)	0.02
329	MBB1A_HUMAN	42	149731	16 (2)	11 (2)	0.04
330	DNJB1_HUMAN	42	38191	4 (2)	3 (2)	0.18
331	SF3B4_HUMAN	41	44414	1 (1)	1 (1)	0.07
332	TBG1_HUMAN	41	51480	6 (1)	4 (1)	0.06
333	B4GT7_HUMAN	41	37838	4 (2)	3 (1)	0.09
334	PLRG1_HUMAN	41	57500	6 (1)	3 (1)	0.06
335	BI2L1_HUMAN	41	57189	3 (1)	3 (1)	0.06
336	OAT_HUMAN	41	48846	2 (1)	2 (1)	0.07
337	CN166_HUMAN	40	28165	3 (2)	3 (2)	0.25
338	GCSAM_HUMAN	40	21334	4 (2)	2 (1)	0.16
339	SMC3_HUMAN	40	141853	14 (2)	12 (2)	0.05
340	MTSSL_HUMAN	40	80450	5 (1)	3 (1)	0.04
341	RL5_HUMAN	40	34569	3 (1)	3 (1)	0.1
342	RPB3_HUMAN	40	31764	2 (1)	2 (1)	0.1
343	PLS1_HUMAN	40	36052	1 (1)	1 (1)	0.09
344	NUD16_HUMAN	40	21317	2 (2)	2 (2)	0.34
345	IMDH2_HUMAN	39	56226	4 (1)	3 (1)	0.06
346	RRAS2_HUMAN	39	23613	2 (1)	2 (1)	0.14
347	RBMX2_HUMAN	39	37541	2 (1)	2 (1)	0.09
348	EIF2D_HUMAN	39	65293	1 (1)	1 (1)	0.05
349	RU17_HUMAN	39	51583	6 (3)	4 (3)	0.2

350	MGP_HUMAN	39	12516	2 (2)	1 (1)	0.27
351	MX2_HUMAN	39	82493	6 (2)	6 (2)	0.08
352	NOP53_HUMAN	39	54470	7 (3)	1 (1)	0.06
353	BAZ1B_HUMAN	38	172392	16 (3)	10 (2)	0.04
354	FLNB_HUMAN	38	280157	17 (3)	14 (3)	0.04
355	KLC1_HUMAN	38	65782	10 (4)	9 (4)	0.22
356	EIF3L_HUMAN	38	66912	8 (2)	8 (2)	0.1
357	WDR5_HUMAN	38	37136	1 (1)	1 (1)	0.09
358	RNPS1_HUMAN	38	34188	5 (1)	4 (1)	0.1
359	SNUT2_HUMAN	38	65739	3 (2)	3 (2)	0.1
360	TFG_HUMAN	38	43478	2 (1)	2 (1)	0.08
361	TECR_HUMAN	38	36410	2 (2)	2 (2)	0.19
362	AP3M1_HUMAN	38	47251	2 (1)	2 (1)	0.07
363	DECR2_HUMAN	38	31100	5 (1)	3 (1)	0.11
364	HDGF_HUMAN	37	26886	5 (1)	4 (1)	0.12
365	NXF1_HUMAN	37	70652	10 (2)	8 (2)	0.1
366	RSU1_HUMAN	37	31521	3 (1)	2 (1)	0.11
367	NAA50_HUMAN	37	19614	1 (1)	1 (1)	0.17
368	SPT4H_HUMAN	37	13470	2 (1)	2 (1)	0.25
369	IBP5_HUMAN	37	31576	9 (1)	6 (1)	0.11
370	DLDH_HUMAN	37	54713	5 (1)	5 (1)	0.06
371	FRMD6_HUMAN	37	72853	4 (1)	4 (1)	0.05
372	SF01_HUMAN	37	68514	2 (1)	2 (1)	0.05
373	PBIP1_HUMAN	37	81050	2 (1)	2 (1)	0.04
374	EIF1B_HUMAN	37	12930	1 (1)	1 (1)	0.27
375	GMPPA_HUMAN	36	46604	3 (1)	2 (1)	0.07
376	CNTP1_HUMAN	36	158220	4 (1)	4 (1)	0.02
377	GAR1_HUMAN	36	22505	1 (1)	1 (1)	0.15
378	NUB1_HUMAN	36	71235	5 (1)	5 (1)	0.05
379	ACSL3_HUMAN	36	81338	6 (1)	5 (1)	0.04
380	ARF5_HUMAN	35	20631	6 (3)	5 (2)	0.57
381	RT34_HUMAN	35	25692	6 (2)	4 (2)	0.28
382	IF4A3_HUMAN	35	47126	7 (1)	6 (1)	0.07
383	WASL_HUMAN	35	55192	10 (1)	5 (1)	0.06
384	WASC5_HUMAN	35	135113	9 (1)	7 (1)	0.02
385	GALT2_HUMAN	35	65433	2 (1)	2 (1)	0.05
386	STX5_HUMAN	35	39762	4 (1)	4 (1)	0.08
387	MYO1B_HUMAN	35	132928	7 (1)	7 (1)	0.02
388	EIF3B_HUMAN	34	92823	6 (1)	4 (1)	0.04
389	MRRP1_HUMAN	34	47602	8 (3)	8 (3)	0.22
390	BRK1_HUMAN	34	8796	3 (1)	3 (1)	0.4
391	TRAM1_HUMAN	34	43215	2 (1)	2 (1)	0.08
392	SP110_HUMAN	33	79600	3 (1)	3 (1)	0.04
393	MK01_HUMAN	33	41762	6 (1)	5 (1)	0.08

394	DDX41_HUMAN	33	70477	9 (1)	9 (1)	0.05
395	RT14_HUMAN	33	15243	3 (1)	3 (1)	0.22
396	C2D1B_HUMAN	33	94394	17 (2)	9 (2)	0.07
397	EFTU_HUMAN	33	49852	4 (2)	4 (2)	0.14
398	TRM6_HUMAN	33	56049	9 (1)	7 (1)	0.06
399	BCKD_HUMAN	33	46616	4 (1)	4 (1)	0.07
400	PLEC_HUMAN	32	533462	49 (2)	39 (2)	0.01
401	PPOX_HUMAN	32	51190	4 (1)	3 (1)	0.06
402	PGLT1_HUMAN	32	46615	9 (1)	7 (1)	0.07
403	TAP1_HUMAN	32	87733	2 (1)	2 (1)	0.04
404	TREX1_HUMAN	32	39525	4 (1)	4 (1)	0.08
405	MACD1_HUMAN	32	35996	4 (1)	4 (1)	0.09
406	KMT2E_HUMAN	32	206891	7 (1)	7 (1)	0.02
407	GARS_HUMAN	31	83854	10 (1)	9 (1)	0.04
408	LMO7_HUMAN	31	194002	7 (1)	6 (1)	0.02
409	SIKE1_HUMAN	31	23877	8 (1)	7 (1)	0.14
410	ANKH1_HUMAN	31	271286	14 (1)	13 (1)	0.01
411	BCR_HUMAN	31	143756	5 (1)	5 (1)	0.02
412	TNAP2_HUMAN	31	73015	10 (3)	8 (3)	0.14
413	PCLO_HUMAN	31	554704	39 (2)	20 (1)	0.01
414	PDK3_HUMAN	31	47080	5 (1)	4 (1)	0.07
415	ERP44_HUMAN	30	47341	3 (2)	3 (2)	0.14
416	RU2B_HUMAN	30	25470	3 (2)	3 (2)	0.28
417	NQO1_HUMAN	30	30905	1 (1)	1 (1)	0.11
418	ARSB_HUMAN	30	60163	2 (1)	2 (1)	0.05
419	KIF2A_HUMAN	30	80589	3 (1)	3 (1)	0.04
420	DDX27_HUMAN	30	90292	5 (2)	5 (2)	0.07
421	ACOX1_HUMAN	30	74889	4 (1)	4 (1)	0.04
422	ASAP2_HUMAN	30	112835	5 (2)	5 (2)	0.06
423	SFR15_HUMAN	30	126132	5 (1)	4 (1)	0.03
424	PARVA_HUMAN	29	42274	6 (1)	6 (1)	0.08
425	XPC_HUMAN	29	106743	8 (1)	8 (1)	0.03
426	OAS3_HUMAN	29	122861	19 (4)	15 (4)	0.11
427	KTN1_HUMAN	29	156464	19 (2)	14 (2)	0.04
428	RS23_HUMAN	29	15969	4 (1)	3 (1)	0.21
429	ANKE1_HUMAN	28	87351	11 (2)	9 (2)	0.08
430	RM48_HUMAN	28	24034	2 (1)	2 (1)	0.14
431	RBP1_HUMAN	28	76415	3 (1)	2 (1)	0.04
432	OBSCN_HUMAN	28	879630	43 (1)	38 (1)	
433	FOSL2_HUMAN	28	35343	4 (1)	3 (1)	0.09
434	GPNMB_HUMAN	28	64623	4 (1)	3 (1)	0.05
435	XRN1_HUMAN	28	195524	13 (1)	11 (1)	0.02
436	YTHD3_HUMAN	28	63936	11 (1)	9 (1)	0.05
437	TAGL2_HUMAN	28	22548	2 (1)	2 (1)	0.15

438	NDK8_HUMAN	28	15690	4 (2)	2 (1)	0.48
439	TIMP1_HUMAN	28	23840	1 (1)	1 (1)	0.14
440	LOXL2_HUMAN	28	88778	3 (1)	3 (1)	0.04
441	SAHH3_HUMAN	28	67705	6 (1)	6 (1)	0.05
442	EWS_HUMAN	28	68721	8 (1)	7 (1)	0.05
443	ABCAD_HUMAN	27	580524	18 (1)	15 (1)	0.01
444	TRI56_HUMAN	27	83147	9 (1)	5 (1)	0.04
445	AAKG1_HUMAN	27	37727	4 (1)	4 (1)	0.09
446	POC1A_HUMAN	27	45551	5 (2)	1 (1)	0.07
447	CFAH_HUMAN	27	143680	2 (1)	2 (1)	0.02
448	BPIB3_HUMAN	27	50539	2 (1)	2 (1)	0.07
449	ERF3A_HUMAN	27	56348	2 (1)	2 (1)	0.06
450	RAF1_HUMAN	27	73803	11 (1)	7 (1)	0.04
451	CAV3_HUMAN	27	17761	2 (1)	2 (1)	0.19
452	PKHA4_HUMAN	27	85577	5 (1)	4 (1)	0.04
453	T132C_HUMAN	27	122624	13 (1)	11 (1)	0.03
454	KPCA_HUMAN	27	77841	7 (1)	7 (1)	0.04
455	DPOE1_HUMAN	27	264031	14 (1)	10 (1)	0.01
456	ARPC5_HUMAN	27	16367	3 (1)	3 (1)	0.21
457	F186A_HUMAN	27	262994	12 (1)	10 (1)	0.01
458	ILK_HUMAN	27	51899	11 (1)	10 (1)	0.06
459	SRRM1_HUMAN	27	102331	10 (1)	10 (1)	0.03
460	GLDN_HUMAN	27	59262	10 (1)	4 (1)	0.06
461	C1TC_HUMAN	26	102180	10 (1)	8 (1)	0.03
462	CMTR1_HUMAN	26	96172	5 (1)	4 (1)	0.03
463	PMGT2_HUMAN	26	67086	2 (1)	2 (1)	0.05
464	KCC2B_HUMAN	26	73544	5 (1)	5 (1)	0.04
465	SMIM1_HUMAN	26	8915	2 (1)	2 (1)	0.39
466	TAF4B_HUMAN	26	91832	5 (1)	3 (1)	0.04
467	UBFL1_HUMAN	26	46447	3 (1)	2 (1)	0.07
468	SRPK2_HUMAN	26	78219	9 (1)	6 (1)	0.04
469	SHIP1_HUMAN	26	134121	8 (1)	7 (1)	0.02
470	EST1_HUMAN	26	62766	2 (1)	2 (1)	0.05
471	CFAB_HUMAN	26	86847	6 (1)	6 (1)	0.04
472	SREK1_HUMAN	26	59402	5 (1)	4 (1)	0.06
473	CHSS1_HUMAN	26	92353	6 (1)	6 (1)	0.04
474	GDPD5_HUMAN	26	69112	2 (1)	2 (1)	0.05
475	SPCS2_HUMAN	26	25272	6 (1)	4 (1)	0.13
476	PLAP_HUMAN	25	88641	10 (1)	6 (1)	0.04
477	U17L1_HUMAN	25	60692	5 (1)	4 (1)	0.05
478	TDRD9_HUMAN	25	157124	10 (1)	6 (1)	0.02
479	DDAH1_HUMAN	25	31444	9 (1)	3 (1)	0.11
480	SLTM_HUMAN	25	117364	7 (1)	7 (1)	0.03
481	TM104_HUMAN	25	56327	2 (1)	2 (1)	0.06

482	DAPLE_HUMAN	25	229231	33 (1)	11 (1)	0.01
483	TLK2_HUMAN	25	88405	6 (1)	6 (1)	0.04
484	KCTD8_HUMAN	25	52863	8 (1)	6 (1)	0.06
485	RPAC1_HUMAN	25	39453	5 (1)	4 (1)	0.08
486	SSH1_HUMAN	25	116465	11 (1)	8 (1)	0.03
487	B3GT6_HUMAN	25	37513	2 (1)	2 (1)	0.09
488	C2C4A_HUMAN	25	40346	5 (2)	3 (1)	0.08
489	ZN180_HUMAN	24	81171	1 (1)	1 (1)	0.04
490	ANR12_HUMAN	24	237046	12 (1)	7 (1)	0.01
491	RBBP7_HUMAN	24	48132	3 (1)	3 (1)	0.07
492	CILP1_HUMAN	24	134761	9 (2)	7 (2)	0.05
493	TARSH_HUMAN	24	119253	10 (2)	8 (2)	0.06
494	SNTB2_HUMAN	24	58369	3 (1)	3 (1)	0.06
495	ZRAB2_HUMAN	24	37838	3 (1)	3 (1)	0.09
496	ACO11_HUMAN	24	69361	8 (1)	6 (1)	0.05
497	CCAR1_HUMAN	24	133423	8 (1)	7 (1)	0.02
498	HYOU1_HUMAN	24	111494	10 (1)	8 (1)	0.03
499	ARHG2_HUMAN	24	112386	7 (1)	7 (1)	0.03
500	DREB_HUMAN	24	71842	2 (1)	2 (1)	0.05
501	SRGP1_HUMAN	23	125099	10 (1)	8 (1)	0.03
502	ZBT47_HUMAN	23	84133	2 (1)	2 (1)	0.04
503	RD23B_HUMAN	23	43202	4 (1)	3 (1)	0.08
504	R51A1_HUMAN	23	38662	10 (1)	6 (1)	0.09
505	DHB12_HUMAN	23	34416	1 (1)	1 (1)	0.1
506	SPD2A_HUMAN	23	125897	11 (1)	9 (1)	0.03
507	ARL2_HUMAN	23	21036	2 (1)	2 (1)	0.16
508	EMC3_HUMAN	23	29932	2 (1)	2 (1)	0.11
509	IF5A1_HUMAN	22	17049	2 (1)	2 (1)	0.2
510	U520_HUMAN	22	246006	10 (1)	8 (1)	0.01
511	BPTF_HUMAN	22	340791	18 (1)	17 (1)	0.01
512	RAB32_HUMAN	22	25210	3 (1)	3 (1)	0.13
513	DJB11_HUMAN	22	40774	5 (1)	4 (1)	0.08
514	PPIL2_HUMAN	22	59243	1 (1)	1 (1)	0.06
515	PDE6B_HUMAN	22	99469	13 (1)	6 (1)	0.03
516	TMED2_HUMAN	22	22860	2 (1)	2 (1)	0.15
517	CF132_HUMAN	22	124414	10 (1)	9 (1)	0.03
518	FCHO2_HUMAN	22	89382	5 (1)	5 (1)	0.04
519	EPHB2_HUMAN	22	119128	21 (1)	5 (1)	0.03
520	DCSTP_HUMAN	21	54099	2 (1)	2 (1)	0.06
521	TSG6_HUMAN	21	31639	3 (1)	3 (1)	0.11
522	KC1E_HUMAN	21	47570	7 (2)	7 (2)	0.14
523	CDK4_HUMAN	21	33936	4 (1)	4 (1)	0.1
524	HTRA2_HUMAN	21	48868	3 (1)	3 (1)	0.07
525	APOA1_HUMAN	21	30759	1 (1)	1 (1)	0.11

526	PRS8_HUMAN	21	45768	4 (1)	4 (1)	0.07
527	CLCKB_HUMAN	21	76024	3 (1)	3 (1)	0.04
528	ZN345_HUMAN	20	57229	1 (1)	1 (1)	0.06
529	TM9S2_HUMAN	20	76809	2 (1)	2 (1)	0.04
530	CFA97_HUMAN	20	59781	5 (1)	4 (1)	0.05
531	RGPS1_HUMAN	20	62550	3 (1)	3 (1)	0.05
532	FRPD2_HUMAN	20	145845	6 (1)	4 (1)	0.02
533	IMB1_HUMAN	20	98420	5 (1)	4 (1)	0.03
534	TIF1B_HUMAN	20	90261	1 (1)	1 (1)	0.04
535	TRA2B_HUMAN	20	33760	5 (1)	4 (1)	0.1
536	UBP32_HUMAN	20	183821	6 (1)	5 (1)	0.02
537	SL9B2_HUMAN	20	58154	3 (1)	2 (1)	0.06
538	KLOTB_HUMAN	19	120473	7 (1)	6 (1)	0.03
539	EIF3M_HUMAN	19	42932	2 (1)	2 (1)	0.08
540	PRC1_HUMAN	19	72246	7 (1)	5 (1)	0.05
541	AL7A1_HUMAN	19	59020	9 (1)	8 (1)	0.06
542	ODB2_HUMAN	19	53852	4 (1)	4 (1)	0.06
543	CATA_HUMAN	19	59947	3 (1)	2 (1)	0.05
544	DHX58_HUMAN	18	77477	2 (1)	2 (1)	0.04
545	PLCB3_HUMAN	18	139511	3 (1)	3 (1)	0.02
546	AKA11_HUMAN	18	212946	6 (1)	6 (1)	0.02
547	SMCA5_HUMAN	18	122513	10 (1)	10 (1)	0.03
548	DNJC9_HUMAN	18	30062	4 (1)	2 (1)	0.11
549	RSLBA_HUMAN	17	27160	1 (1)	1 (1)	0.12
550	CDRT4_HUMAN	17	17617	2 (1)	1 (1)	0.19
551	CPXM1_HUMAN	17	82243	3 (1)	3 (1)	0.04
552	SEPT5_HUMAN	16	43206	5 (1)	5 (1)	0.08
553	THUM3_HUMAN	16	57765	5 (1)	5 (1)	0.06
554	D11L8_HUMAN	16	102944	11 (1)	7 (1)	0.03
555	HINT1_HUMAN	16	13907	1 (1)	1 (1)	0.25
556	SMC2_HUMAN	16	136085	11 (1)	10 (1)	0.02
557	XRP2_HUMAN	16	40471	1 (1)	1 (1)	0.08
558	ESPL1_HUMAN	15	236564	10 (1)	8 (1)	0.01
559	NFXL1_HUMAN	15	107713	6 (1)	6 (1)	0.03
560	CI084_HUMAN	15	167207	10 (1)	5 (1)	0.02
561	AJUBA_HUMAN	15	58722	6 (1)	5 (1)	0.06
562	SMAD3_HUMAN	15	48905	4 (1)	3 (1)	0.07
567	THIOM_HUMAN	15	18543	8 (1)	2 (1)	0.18

Abbreviations: Score, protein scores; Mass, molecular weight of matched protein; Matches, figures in brackets indicate the number of matched peptides (*P<0.05*); Sequences, type of peptide segment sequences which matches the respective peptide (*P<0.05*), emPAI, index of protein abundance.
