# Supplementary material for: Long Non-coding RNA HOTTIP Promotes CCL3 Expression and Induces Cartilage Degradation by Sponging miR-455-3p
Source: Front Cell Dev Biol. 2019 Aug 23;7:161. doi: 10.3389/fcell.2019.00161 (PMC6716540; doi:10.3389/fcell.2019.00161)
Supplement: Supplementary file 3 [file Image_1.pdf]

Supplementary Figure 1. Effect of miR-455-3p and HOTTIP on IL-1β-induced chondrocyte responses.

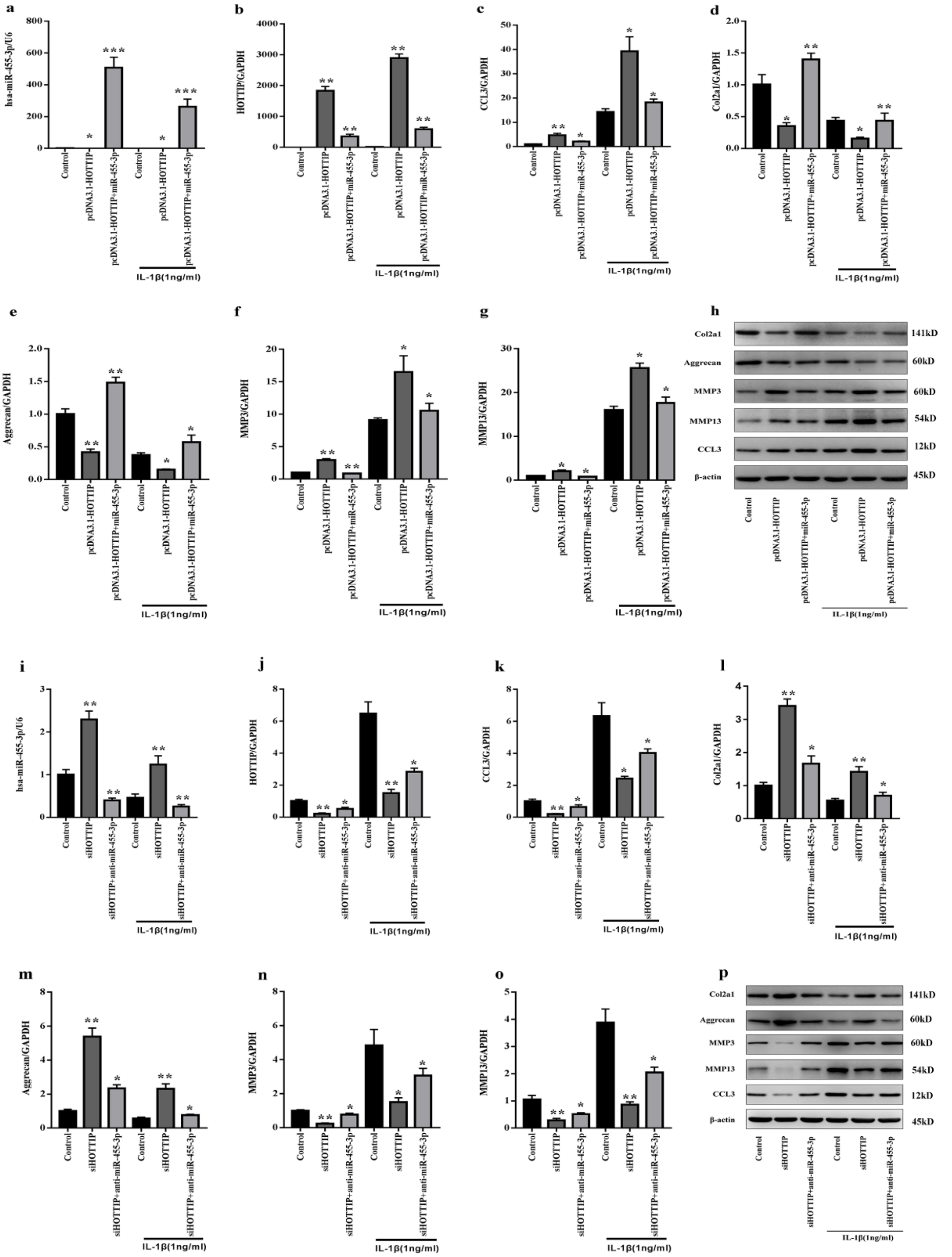

PHCs were transfected with control, pcDNA3.1-HOTTIP, miR-455-3p+ pcDNA3.1-HOTTIP, siNC, siHOTTIP, siHOTTIP+anti-miR-455-3p, then left unstimulated or stimulated with IL-1β for 24 h for RT-qPCR analysis or for 48 h

protein analysis, respectively. The expression level of miR-455-3p (a, i) and HOTTIP (b, j) were estimated by RT-qPCR, while the expression levels of CCL3 (c, h, k, p), COL2A1 (d, h, l, p), aggrecan (e, h, m, p), MMP3 (f, h, n, p), and MMP13 (g, h, o, p) were estimated by RT-qPCR and western blotting. U6, GAPDH, and β-actin were used as endogenous controls. The data shown represent the mean ± SD of at least three independent experiments.*$P < 0.05$, **$P < 0.01$, ***$P < 0.001$.
